# Transcriptome analysis reveals defense-related genes and pathways during dodder (*Cuscuta australis*) parasitism on white clover (*Trifolium repens*)

**DOI:** 10.3389/fgene.2023.1106936

**Published:** 2023-03-16

**Authors:** Li Zhou, Alexander Zawaira, Qiuwei Lu, Beifen Yang, Junmin Li

**Affiliations:** ^1^ Zhejiang Provincial Key Laboratory of Plant Evolutionary Ecology and Conservation, Taizhou University, Taizhou, Zhejiang, China; ^2^ School of Advanced Study, Taizhou University, Taizhou, Zhejiang, China

**Keywords:** parasitic plant dodder, transcriptome, plant hormone, transcription factors, lignin

## Abstract

Dodders (*Cuscuta australis* R. Br.) are holo-parasitic stem angiosperms with an extensive host range that have significant ecological and economic potential impact on the ecosystem and the agricultural system. However, how the host plant responds to this biotic stress remains mostly unexplored. To identify the defense-related genes and the pathways in white clover (*Trifolium repens* L.) induced by dodder parasitism, we performed a comparative transcriptome analysis of the leaf and root tissues from white clover with and without dodder infection by high throughput sequencing. We identified 1,329 and 3,271 differentially expressed genes (DEGs) in the leaf and root tissues, respectively. Functional enrichment analysis revealed that plant-pathogen interaction, plant hormone signal transduction, and phenylpropanoid biosynthesis pathways were significantly enriched. Eight WRKY, six AP2/ERF, four bHLH, three bZIP, three MYB, and three NAC transcription factors showed a close relationship with lignin synthesis-related genes, which defended white clover against dodder parasitism. Real-time quantitative PCR (RT-qPCR) for nine DEGs, further validated the data obtained from transcriptome sequencing. Our results provide new insights into understanding the complex regulatory network behind these parasite-host plant interactions.

## Introduction

*Cuscuta* spp. (family Convolvulaceae), commonly known as dodders, are obligate holoparasites with limited photosynthesis (Revill et al., 2005), that acquire all their nutrients and water from host plants *via* haustoria (Dawson et al., 1994; KC, 2002; Runyon et al., 2006). With a broad host range of crops, *Cuscuta* spp. have been among the most devastating agricultural pests, damaging the worldwide annual output of agricultural crops by millions of dollars (Press & Phoenix, 2005; Bardgett et al., 2006; Zagorchev et al., 2021a). White clover (*Trifolium repens* L.) is a preferred host of *C. australis* in the field (Yu et al., 2019). Understanding the molecular mechanisms underlying the interaction between *Cuscuta* spp. and their host plants would provide the technical foundation for the effective management of these crop pests.

Since *Cuscuta* spp. could adversely affect host plants like herbivores and pathogens, some reports have shown that the host plants defend against parasitic plants very similarly to those induced by herbivores and pathogens (Pennings & Callaway, 2002; Runyon et al., 2010a; Runyon et al., 2010b). Runyon et al. found that parasitism by *C. pentagona* sequentially induced the jasmonic acid (JA) and salicylic acid (SA) defense pathways in tomato. JA peaked 12 h before SA, suggesting that the defense response is coordinated by the sequential induction of both phytohormones (Runyon et al., 2010b). During caterpillar feeding on dodder, the host JA pathway regulates the transcriptomic changes both in dodder and the host plant (Qin et al., 2019). The contact and penetration of the haustoria, removal of the resources from the host, and the subsequent hormonal changes trigger the host defense responses (Saric-Krsmanovic et al., 2020; Zagorchev et al., 2021b). The host plants’ response to the *Cuscuta* spp. infection includes a hypersensitive-like response (HLR) and the expression of pathogenesis-related (PR) genes (Bringmann et al., 1999; Borsics & Lados, 2002). Most of the plant disease resistance genes (*R* genes) encode nucleotide-binding site leucine-rich repeat (NBS-LRR) proteins, which can be further subdivided into TIR-NB-LRR proteins (TNLs) and CC-NB-LRR proteins (CNLs) (McHale, 2006; Kourelis & van der Hoorn, 2018). They are involved in the plant immune responses to attacks by invaders, including viruses, bacteria, oomycetes, fungi, nematodes, herbivores, and parasitic plants. The first receptor (*CUSCUTA RECEPTOR 1*, *CuRe 1*), an LRR-receptor-like serine/threonine-protein kinase (RLP) was identified in tomato, which initiates the pathogen-associated molecular pattern (PAMP)-triggered immunity of tomato against *C. reflexa* (Hegenauer andKaiser, 2016; Hegenauer et al., 2020). Based on the comparative transcriptomics of dodder-resistant and -susceptible tomato cultivars, it was found that the *Cuscuta R-gene for Lignin-based Resistance 1* (*CuRLR1*, a CC-NBS-LRR) functioned as a receptor for recognizing *C. campestris* signals or effectors, resulting in lignification-based resistance (Jhu et al., 2022).

Secondary metabolites are vital in the plant’s environmental adaption and act as defense compounds against abiotic and biotic stress (Ramakrishna & Ravishankar, 2011; Dhakshinamoorthy et al., 2014). Phenolic secondary metabolites like phenols and lignin are important in the banana’s defense mechanisms against the nematode *Radopholus similis*. The phenol content in nematode-infected plants was twice the amount in the uninfected plants at 3 weeks post infection, due to the biosynthesis and accumulation of secondary metabolites at the infection sites (Dhakshinamoorthy et al., 2014). Lignin, a heterogeneous polymer of aromatic subunits derived from phenylalanine, primarily comprised three major monomers: p-hydroxyphenyl (H), guaiacyl (G), and syringyl (S) monolignols that are synthesized *via* the phenylpropanoid pathway (Raes et al., 2003). Lignification has long been believed as a host defense mechanism against pathogens (Vance CP et al., 1980; Bhuiyan et al., 2009; Bellincampi et al., 2014). In cotton, the *GhLAC15* expression enhanced *Verticillium wilt* resistance by increasing the defense-induced cell wall lignification (Zhang et al., 2019). The lignin accumulation in plant roots and stems reportedly confer host resistance against parasitic plants (Pérez-de-Luque et al., 2007; Jhu et al., 2022). For example, the host lignin composition affects haustorium induction in the root parasitic plants *Striga hermonthica* and *Phtheirospermum japonicum* (Cui et al., 2018). A recent report showed that the resistant tomato cultivar Heinz presented a lignin-based resistance against the stem parasite field dodder (*C. campestris*) infection (Jhu et al., 2022). Additionally, the three transcription factors, *MYB55, WRKY16,* and *Lignin Induction Factor 1* (*LIF1*, an *AP2-like* transcription factor) are important in the lignin biosynthesis (Jhu et al., 2022).

Herbivores-attacking induce defense responses not only in the attacked (local) tissues but also in the other non-attacked distal (systemic) plant parts, indicating that a systemic signal is induced in the herbivores-damaged tissues and the signal can be translocated to the other parts of the whole plant to activate defense (Wu & Baldwin, 2010; Zhuang et al., 2018). For example, above-ground chewing herbivores (caterpillar and weevil) and sucking herbivore (aphid) feeding on leaves not only induced above-ground defense response metabolites (quercetin, quercitrin, isoquercetin, and kaempferitrin) in leaves but also induced below-ground defense chemicals in roots (Xiao et al., 2019). Stronger metabolic changes were found in the apex and root tissues than in undamaged leaflets, indicating rapid and significant whole-plant responses to leaves damage caused by caterpillars (Adam D. Steinbrenner et al., 2011). *Cuscuta campestris* has been proven to transfer N-systemic signals between hosts grown in N-heterogeneous soil, resulting in large transcriptome and DNA methylome changes in the recipient hosts (Zhang et al., 2021). Whether the stem parasitic dodder parasitism of the host stem elicits a defense response from the host roots is largely unknown. In the current study, we analyzed the DEGs involved in the plant hormone signal transduction, plant-pathogen interaction, and phenylpropanoid biosynthesis pathways, and also including the R genes and transcription factors of both the roots and leaves of white clover after dodder parasitism, based on transcriptome sequencing. The results will provide important information for our understanding of how host plants respond to parasitic plants.

## Materials and methods

### Plant materials and treatment

The seeds of white clover (*T. repens* L.) and dodder (*Cuscuta australis*) were collected from Luohe Park, Linhai, Taizhou City, Zhejiang Province, China. White clover seeds were sown in pots filled with peat soil, sand, and vermiculite (2:2:1) and grown in a walk-in climate chamber kept at 25°C/20°C in day (16 h)/night (8 h) conditions. Dodder seeds were sown into peat soil. A 10–15-cm-long dodder seedling was wound counterclockwise around the white clover stems to allow the parasite to infect the host plants. Three pots were infected with dodder and the other three pots were set as controls that without dodder infection. Leaf and root tissues of the white clover were sampled after 2 weeks. Leaves or roots from nine white clover plants were pooled as one biological replicate. Three biological replicates were collected for each: (a) parasitized leaves (designated P_leaf1_, P_leaf2_, and P_leaf3_), (b) control leaves (designated C_leaf1_, C_leaf2_, and C_leaf3_), (c) parasitized roots (designated P_root1_, P_root2_, and P_root3_) and (d) control roots (designated C_root1_, C_root2_, and C_root3_). All collected samples were immediately frozen in liquid nitrogen and then stored at −80°C for further analyses.

### RNA extraction and transcriptome sequencing

Three biological replicates each were taken for leaf and root samples. Total RNA was extracted using a TRIzol Kit (Invitrogen, Carlsbad, CA, United States) according to the manufacturer’s instructions. The concentration, fragment size, and the total RNA integrity were measured using an Agilent 2,100 platform (Agilent Technologies Inc., Santa Clara, CA, United States). The degradation and contamination of total RNA were assessed by 1.2% agarose gel electrophoresis. The leaf and root cDNA libraries were generated using the NEBNext^®^ Ultra™ RNA Library Prep Kit for Illumina^®^ (NEB, United States) and the resulting cDNAs libraries were sequenced on an Illumina Hiseq platform and the paired-end reads were generated (Novogene, China).

### Transcriptome data analysis

Firstly, adapters, poly-N, and low-quality reads were removed from raw reads to generate clean reads. Simultaneously, Q20, Q30, and the GC-content of the clean reads were calculated. All the downstream analyses were based on clean reads with high quality. Then, clean reads were used for transcriptome *de novo* assemble using Trinity (v2.4.0) with min_kmer_cov set to 2 as default along with all the other parameters being set as default, which provided a reference sequence for the downstream analysis (Grabherr et al., 2011). The longest transcripts in the cluster units were regarded as unigenes to eliminate redundant sequences. The function of all the unigenes were annotated based on seven databases: NCBI non-redundant protein sequences (Nr), Clusters of orthologous groups of proteins (KOG), A manually annotated and reviewed protein sequence database (Swiss-Prot), NCBI non-redundant nucleotide sequences (Nt), Gene Ontology (GO), Kyoto Encyclopedia of Genes and Genomes (KEGG), and Protein family (Pfam). Clean reads for every leaf and root sample were mapped to the assembled reference sequence by RSEM (v1.2.15) with bowtie2 set to mismatch 0 as default (Li & Dewey, 2011), resulting in the read count of each unigene in every leaf and root samples, respectively. The expression levels of each unigene were normalized based on the fragments per kilobase of transcript per million mapped reads (FPKM). DEGs in the P_leaf_ vs. C_leaf_ and P_root_ vs. C_root_ were analyzed using the DESeq R package (1.12.0) (Love et al., 2014) with the thresholds of padj <0.05 and the absolute value of log2 (parasitized/unparasitized) > 1. Gene Ontology (GO) enrichment analysis of the DEGs was performed using the GOseq R packages based Wallenius non-central hypergeometric distribution (Young et al., 2010). GO terms with *p*-value <0.05 were considered as significantly enriched. Pathway enrichment of DEGs was performed using the KEGG database (Kanehisa et al., 2008). Then, the KOBAS software (v2.0.12) was used to test the statistical enrichment of the DEGs in the KEGG pathways (Mao et al., 2005). The pathways with *p*-value <0.05 were considered as significantly enriched.

### Transcription factors (TFs) identification and analysis

Transcription factors were identified based on the DEGs using the iTAK software (1.2) (Zheng et al., 2016) with the plant TFDB (Jin et al., 2017). The Pearson’s correlation coefficients between TFs and genes were calculated and the correlation network was visualized using the Cytoscape software (version 3.8.2) (Shannon et al., 2003).

### RT-qPCR validation of the transcriptome data

To validate the reliability of the transcriptome data, nine DEGs that enriched to the main pathways like “plant hormone signal transduction,” “plant–pathogen interaction,” and “starch and sucrose metabolism” were selected for expression analysis by RT-qPCR. The RNA samples used for RT-qPCR were consistent with those for RNA-seq. Total RNA was reversed transcribed into cDNA using a Fastking RT kit (with gDNase; Tiangen, Beijing, China). Then, qPCR was carried out on a CFX Connect Real-time system (Bio-Rad, United States) using a FastFire qPCR premix (SYBR Green) kit (Tiangen, Beijing, China). The qPCR procedure was set as follows: 95°C for 1 min, and 40 cycles of 95°C for 5 s and 60°C for 15 s. *EF1* was used as the reference gene (Wang et al., 2020). Three biological and technical replicates each were performed for the leaf and root samples. The relative expression levels of DEGs were calculated using the 2^−ΔΔCT^ method (Livak & Schmittgen, 2001). The primers used for RT-qPCR were designed by primer premier 6 and the primers are listed in Supplementary Table S1.

## Results

### General analysis of white clover transcriptome data

To further improve our understanding of the molecular response of white clover to dodder parasitism, we performed a comparative transcriptome analysis of the dodder-infected and uninfected root and leaf samples. Three biological replicates from each treatment resulted in 12 cDNA libraries. The transcriptome sequencing generated 2,096 and 2,235 million raw reads in the leaf and root samples, respectively (Supplementary Table S2). After removing the adapter sequences and the low-quality sequences, we retained 27.14 and 28.92 Gb clean data for subsequent analyses in the leaf and root samples, respectively. Quality assessment of the transcriptome data showed that the error rate was very low (0.01%), while it was very high (>97%) for the Q30, thereby indicating a high-quality transcriptome data being generated in this study. Then, all the clean reads were assembled using Trinity, thus generating 288,139 transcripts (N50: 1,420) with a mean length of 1,047 bp (Supplementary Figure S1A). Clustering resulted in 101,498 unigenes (N50: 1,253) with a mean length of 910 bp; while the length of the unigenes ranged from 301 to 106,769 bp (Supplementary Figure S1B). To functionally annotate the assembled unigenes, we compared using a sequence similarity against seven protein databases using BLASTX. As a result, we annotated 77,231 unigenes (76.09%) in at least one database, and 7,981 unigenes (7.86%) in all the other databases (Supplementary Figure S1C). According to NR annotation, we compared 24.2% of the annotated unigenes to *Trifolium pretense*, 21.7% to *T. subterraneum*, 16.9% to *Quercus suber*, 12.2% to *Mesicago truncatula*, 4.4% to *Cicer arietinum*, and 20.5% to other species (Suppementary Figure S1D). All the RNA sequencing raw data can be found in the NCBI BioProject database under accession number PRJNA700502 and PRJNA828029.

### DEGs in response to dodder parasitism

**FIGURE 1:**
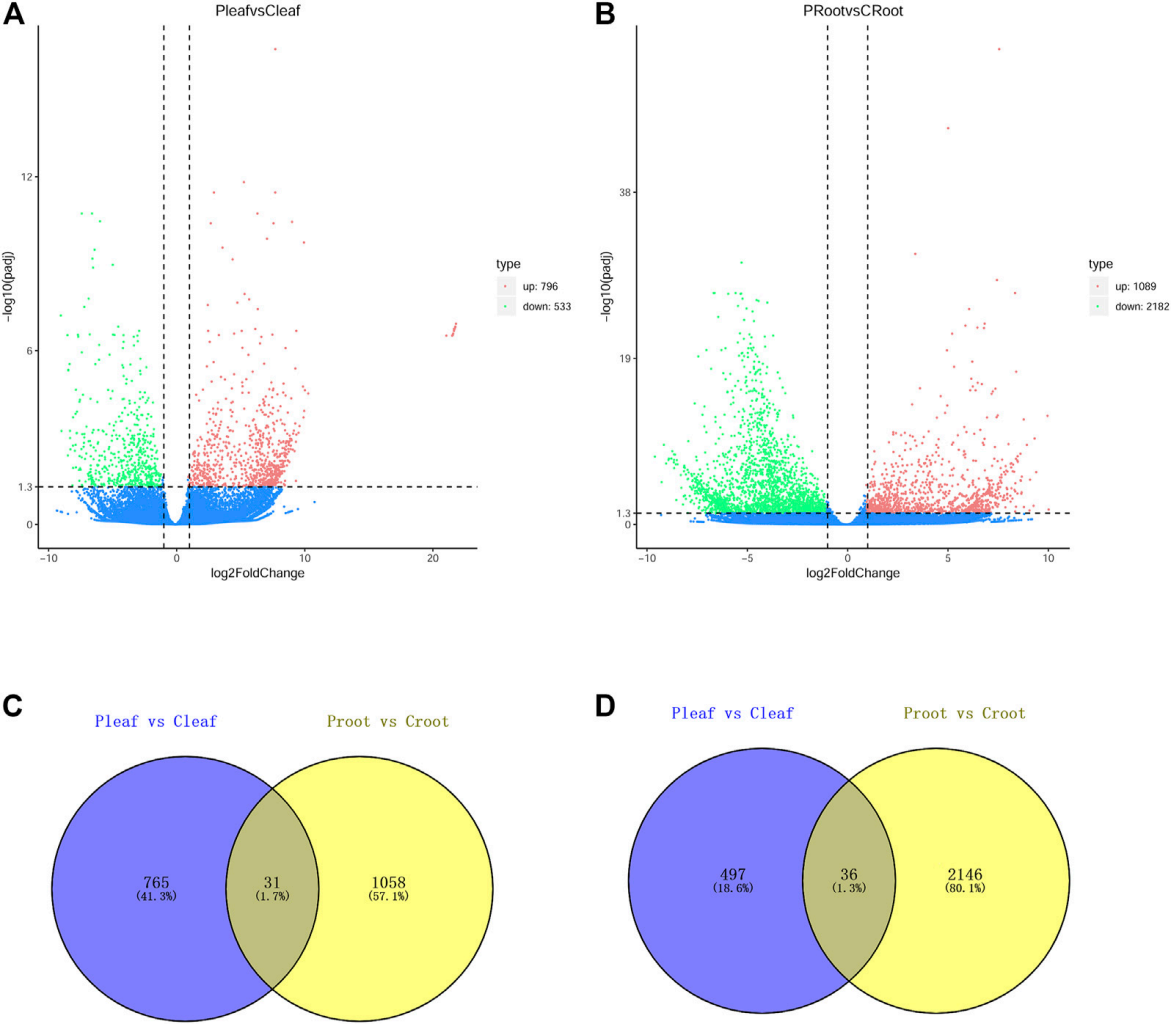
DEGs in leaves and roots of white clover with dodder parasitism. **(A)** Volcano plots displaying the up- and downregulated DEGs in Pleaf vs. Cleaf; **(B)** Volcano plots displaying the up- and downregulated DEGs in Proot vs. Croot; **(C)** Venn diagram showing the shared and unique upregulated DEGs between Pleaf vs. Cleaf and Proot vs. Croot; **(D)** Venn diagram showing the shared and unique downregulated DEGs between Pleaf vs. Cleaf and Proot vs. Croot.

The DEGs were determined with the criteria of |log 2 (FoldChange)| > 1 and padj <0.05. We identified 1,329 DEGs in the leaves from white clover with dodder parasitism, of which 796 and 533 DEGs were up- and downregulated, respectively (Figure 1A). We identified a higher number of DEGs (3,271) in the roots of white clover with dodder parasitism, of which 1,089 and 2,182 were up- and downregulated, respectively (Figure 1B). A venny diagram showed that 31 and 36 DEGs were common in up- and downregulated DEGs in leaves and roots, respectively (Figures 1C, D).

### Identification of the R genes in response to dodder parasitism

Among the upregulated DEGs in the P_leaf_ vs. C_leaf_, we identified 2 R genes, 6 protein kinase genes, 7 PR genes, 17 ubiquitin pathway genes, and 11 calcium signaling genes (Supplementary Table S3). While we identified 49 R genes, 108 protein kinase genes, 6 PR genes, 9 ubiquitin pathway genes, and 11 calcium signaling genes in the downregulated DEGs (Supplementary Table S4). In P_root_ vs. C_root_, we identified 64 R genes, 45 protein kinase genes, 14 PR genes, 14 ubiquitin pathway genes, and 18 calcium signaling genes among the upregulated DEGs (Supplementary Table S5), while we found 22 R proteins, 39 protein kinase genes, 61 PR genes, 55 ubiquitin pathway genes, and 44 calcium signaling genes among the downregulated DEGs (Supplementary Table S6).

### GO and KEGG enrichment analysis of DEGs

To know the putative function classification and pathways of the DEGs, we performed GO enrichment analysis and KEGG pathway enrichment analysis, respectively. A total of 1836 GO terms were enriched based on the upregulated DEGs in P_leaf_ vs. C_leaf_, of which 188 terms were significantly enriched (*p*-value <0.05; Supplementary Figure S2). In the biological process (BP) category, the DEGs were mainly enriched in metabolic process and biosynthetic process, including metabolic process (GO:0008152), cellular process (GO:0009987), organic substance metabolic process (GO:0071704), primary metabolic process (GO:0044238), and cellular metabolic process (GO:0044237). In the cellular component (CC) category, the DEGs were significantly enriched in cell (GO:0005623), cell part (GO:0044464), intracellular (GO:0005622) and intracellular part (GO:0044424), macromolecular complex (GO:0032991), cytoplasm (GO:0005737), and cytoplasmic part (GO:0044444). In the molecular function (MF) category, the DEGs were significantly enriched in structural molecule activity (GO:0005198), structural constituent of ribosome (GO:0003735) and oxidoreductase activity (GO:0016491; Supplementary Figure S2A). A total of 1,297 terms were enriched based on the downregulated DEGs in leaves, of which 88 terms were significantly enriched, including 48 MF, 35 BP and 5 CC (Supplementary Figure S2B). A total of 1792 GO terms were enriched based on the upregulated DEGs in P_root_ vs. C_root_, of which 101 terms were significantly enriched. The upregulated genes were mostly enriched in BP, including response to stimulus (GO:0050896), cell communication (GO:0007154), signal transduction (GO:0007165), single organism signaling (GO:0044700), signalng (GO:0023052), DNA integration (GO:0015074) and DNA metabolic process (GO:0006259; Supplementary Figure S2C). Eighty-five terms were significantly enriched on the downregulated DEGs in roots, including 39 BP, 39 MF and 7 CC. In BP, 457genes were enriched in nodulation (GO:0009877) and nodulation morphogenesis (GO:0009878; Supplementary Figure S2).

**FIGURE 2:**
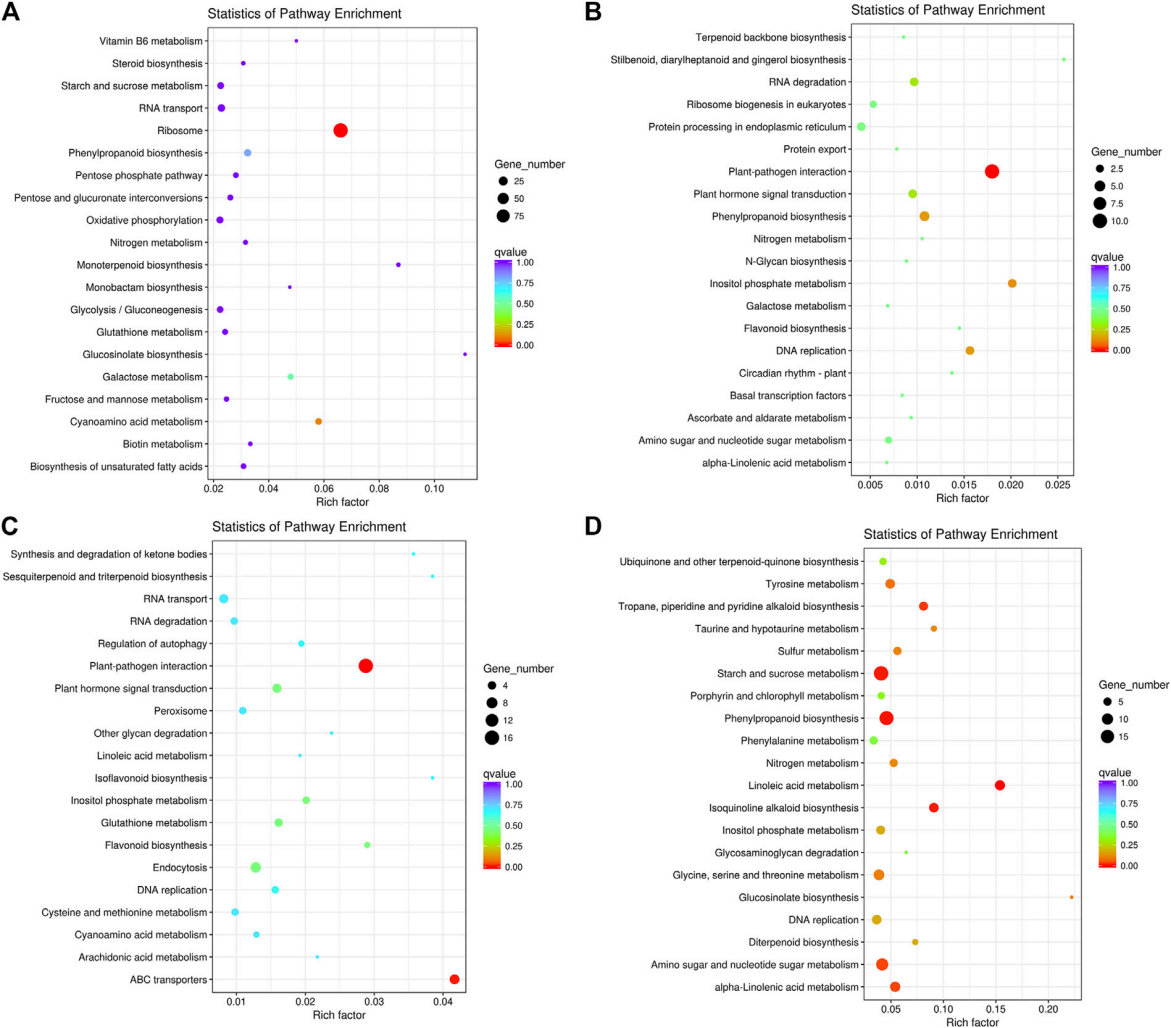
The top 20 enriched KEGG pathways of the DEGs in white clover with dodder parasitism. **(A)** upregulated DEGs in leaves; **(B)** downregulated DEGs in leaves; **(C)** upregulated DEGs in roots; **(D)** downregulated DEGs in roots. The Rich factor is the ratio of the number of differentially expressed genes annotated in this pathway to the number of all genes annotated in this pathway. The higher the Rich factor, the greater the degree of pathway enrichment. A q-value is an adjusted *p*-value; taking into account the false discovery rate and range from 0 to 1 and a lower value indicates greater pathway enrichment.

We also subjected the DEGs to KEGG pathway enrichment analysis. In P_leaf_ vs. C_leaf_, the upregulated DEGs were mainly enriched in ribosome (ko03010), cyanoamino acid metabolism (ko00460), galactose metabolism (ko00052) and phenylpropanoid biosynthesis (ko00940; Figure 2A), while the downregulated DEGs were significantly enriched in plant-pathogen interaction (ko04626), inositol phosphate metabolism (ko00562), DNA replication (ko03030) and phenylpropanoid biosynthesis (ko00940; Figure 2B). In P_root_ vs. C_root_, the upregulated DEGs were significantly enriched in plant-pathogen interaction (ko04626), ABC transporters (ko02010), plant hormone signal transduction (ko04075) and endocytosis (ko04144; Figure 2C), while the downregulated DEGs were significantly enriched in linoleic acid metabolism “(ko00591), phenylpropanoid biosynthesis (ko00940), starch and sucrose metabolism (ko00500), isoquinoline alkaloid biosynthesis (ko00950), tropane, piperidine and pyridine alkaloid biosynthesis (ko00960), amino sugar and nucleotide sugar metabolism (ko00520), alpha-Linolenic acid metabolism (ko00592), tyrosine metabolism (ko00350), glucosinolate biosynthesis (ko00966), glycine, serine and threonine metabolism (ko00260), sulfur metabolism (ko00920), taurine and hypotaurine metabolism (koko00430), nitrogen metabolism (ko00910), diterpenoid biosynthesis (ko00904), inositol phosphate metabolism (ko00562) and DNA replication (ko03030; Figure 2D).

### DEGs related to plant-pathogen interaction pathway

**FIGURE 3:**
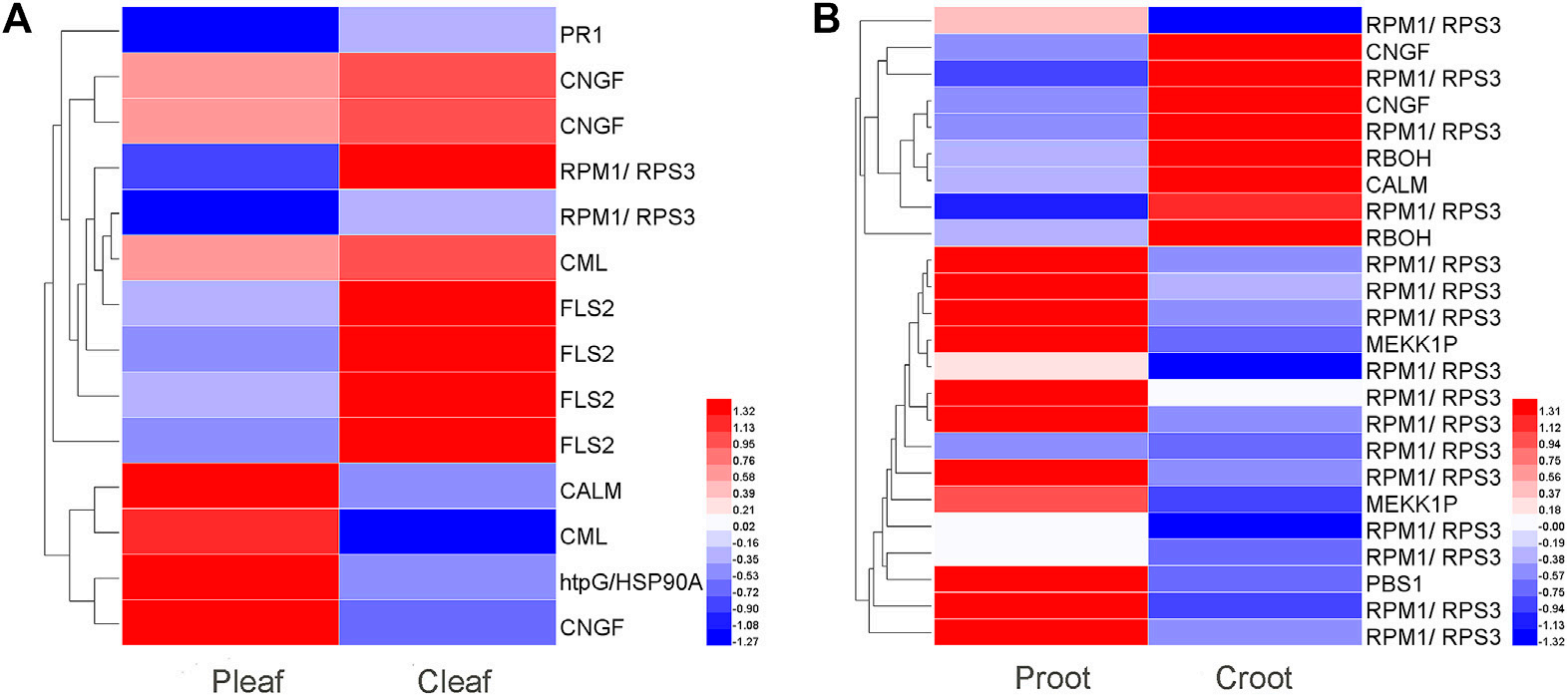
DEGs enriched in the plant–pathogen interaction pathway in white clover after dodder parasitism. **(A)** Leaves; **(B)** Roots.

We identified 14 and 24 DEGs related to plant-pathogen interaction in the leaves and roots, respectively (Figure 3 and Supplementary Table S7). In P_leaf_ vs. C_leaf_, most of the DEGs (10 DEGs) were downregulated, including one *PR1*, two *CNGF*, two *RPM1/RPS3*, one *CML,* and four *FLS2*, while only four DEGs were upregulated, including *CALM*, *HtpG/HSP90A*, *CML,* and *CNGF* (Figure 3A). Contrarily, more DEGs (16 DEGs) were upregulated in the P_root_ vs. C_root_, including 13 *RPM1/RPS3*, two *MEKK1P*, and one *PBS1*, while only eight DEGs were downregulated, including three *RPM1/RPS3*, two *CNGF*, two *RBOH,* and one *CALM* (Figure 3B).

### DEGs related to plant hormone signal transduction pathway

**FIGURE 4:**
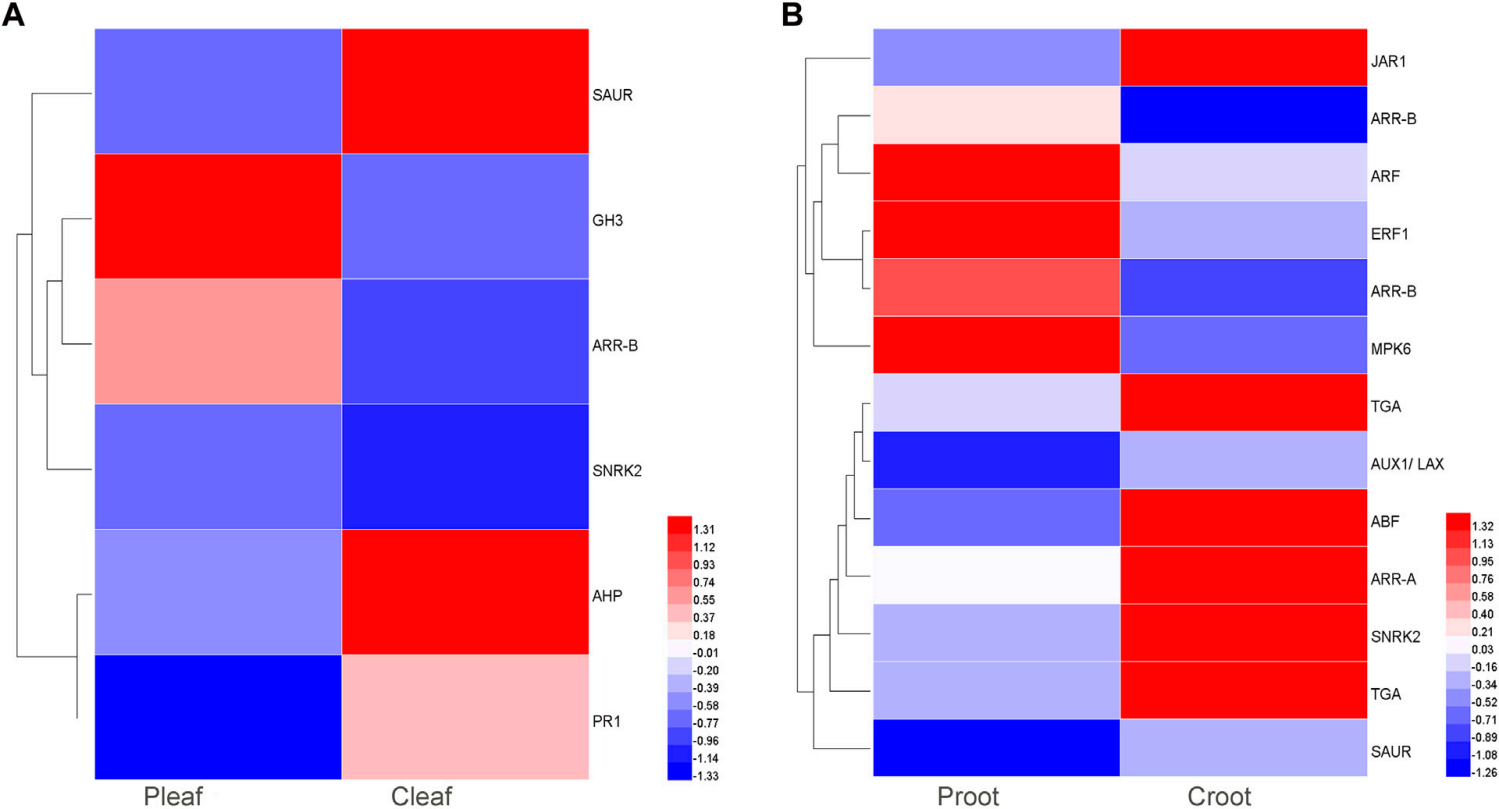
DEGs enriched in the plant hormone signal transduction pathway in white clover after dodder parasitism. **(A)** Leaves; **(B)** Roots.

We detected 6 and 13 DEGs to be involved in plant hormone signal transduction in the leaves and roots, respectively. These DEGs were related to various phytohormones like abscisic acid (ABA), auxin, cytokinin (CTK), ethylene (ET), salicylic acid (SA), and jasmonic acid (JA) (Figure 4 and Supplementary Table S8). ABA, auxin, CTK, and SA related genes were differentially expressed in both P_leaf_ vs. C_leaf_ and P_root_ vs. C_root_, while ET and JA related genes were specific to the P_root_ vs. C_root_. Specifically, three DEGs were involved in the ABA metabolism, including one upregulated *SNRK2* (Cluster-28897.34756) gene in the leaves and two downregulated genes, *ABF* (Cluster-28897.5726) and *SNRK2* (Cluster-57825.0) in the roots. In the leaf samples, we found that two DEGs involved in auxin metabolism or signaling were significantly affected by the dodder infection, with the *GH3* (Cluster-28897.5110) and *SAUR* (Cluster-40615.0) genes being significantly upregulated and downregulated, respectively. In the root samples, three DEGs were related to auxin signaling, including one upregulated *ARF* (Cluster-28897.37861) gene, and two downregulated genes, *AUX1* (Cluster-45618.0) and *SAUR* (Cluster-28897.6014). Three SA related DEGs (one *PR1* and two *TGA*) were both downregulated in leaves and roots. The specific JA related DEG *JAR1* was also downregulated in roots.

### DEGs related to phenylpropanoid biosynthesis pathway

**FIGURE 5:**
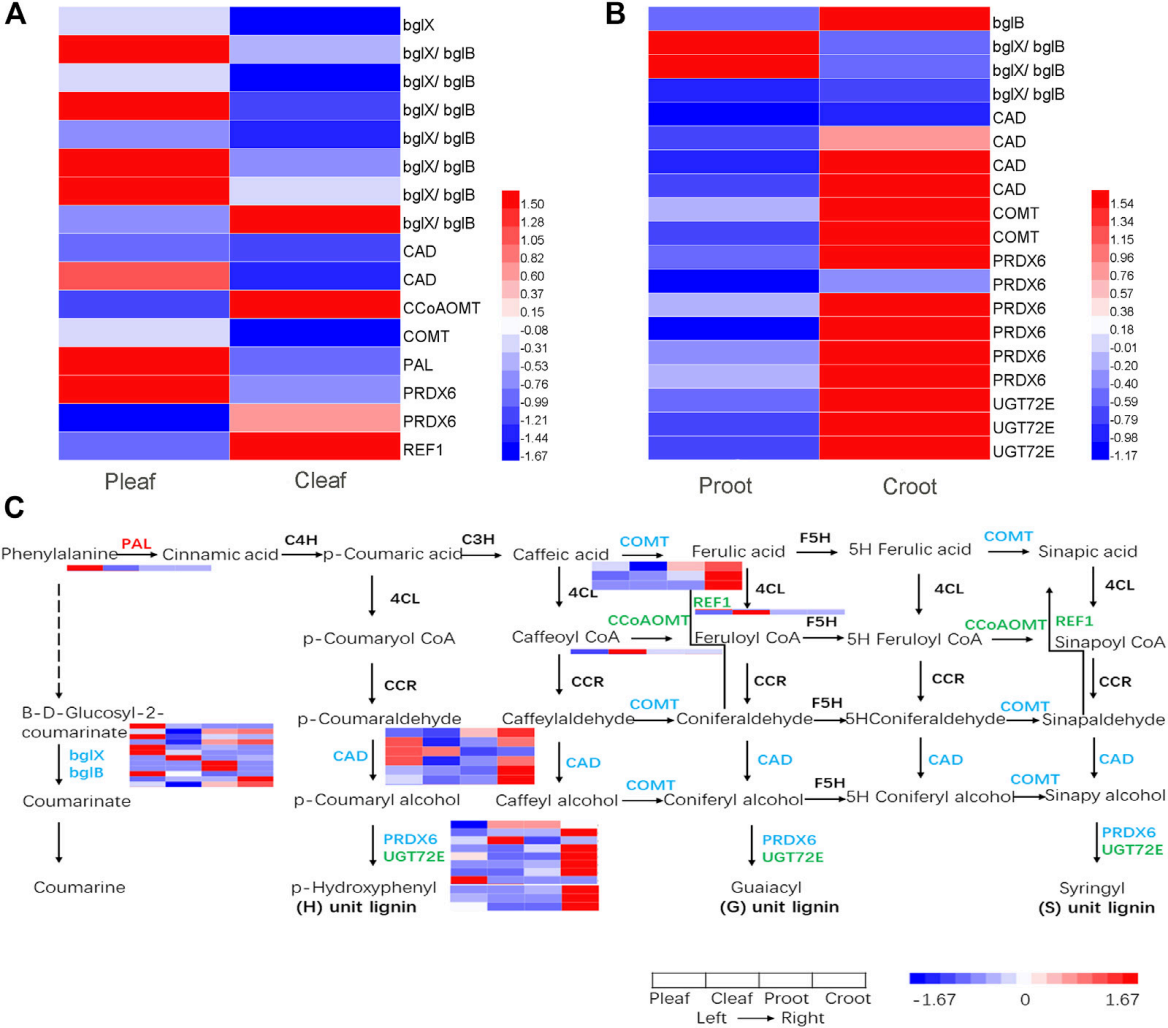
DEGs enriched in the phenylpropanoid biosynthesis pathway in the **(A)** leaves and **(B)** roots from white clover after dodder parasitism. **(C)** Pathway of phenylpropanoid biosynthesis. The red, green, and blue colorations of the gene names indicate upregulated, downregulated, and both upregulated and downregulated, respectively.

We detected 16 and 19 DEGs to be involved in phenylpropanoid biosynthesis in the leaves and roots, respectively (Figures 5A, B and Supplementary Table S9). Among these DEGs, 10 were structural enzymes in the coumarine biosynthesis pathway. The other 25 DEGs were structural enzymes closely involved in the lignin biosynthesis, including one phenylalanine ammonia-lyase (PAL), three caffeic acid 3-O-methyltransferase (COMT) genes, one caffeoyl-CoA O-methyltransferase (CCoAOMT) gene, one coniferyl-aldehyde dehydrogenase (REF1) gene, six cinnamyl-alcohol dehydrogenase (CAD) genes, eight peroxidase (PRDX6) genes, and three coniferyl-alcohol glucosyltransferase (UGT72E) genes (Figure 5C).

### Differentially expressed transcription factors (TFs) in response to dodder parasitism

**FIGURE 6:**
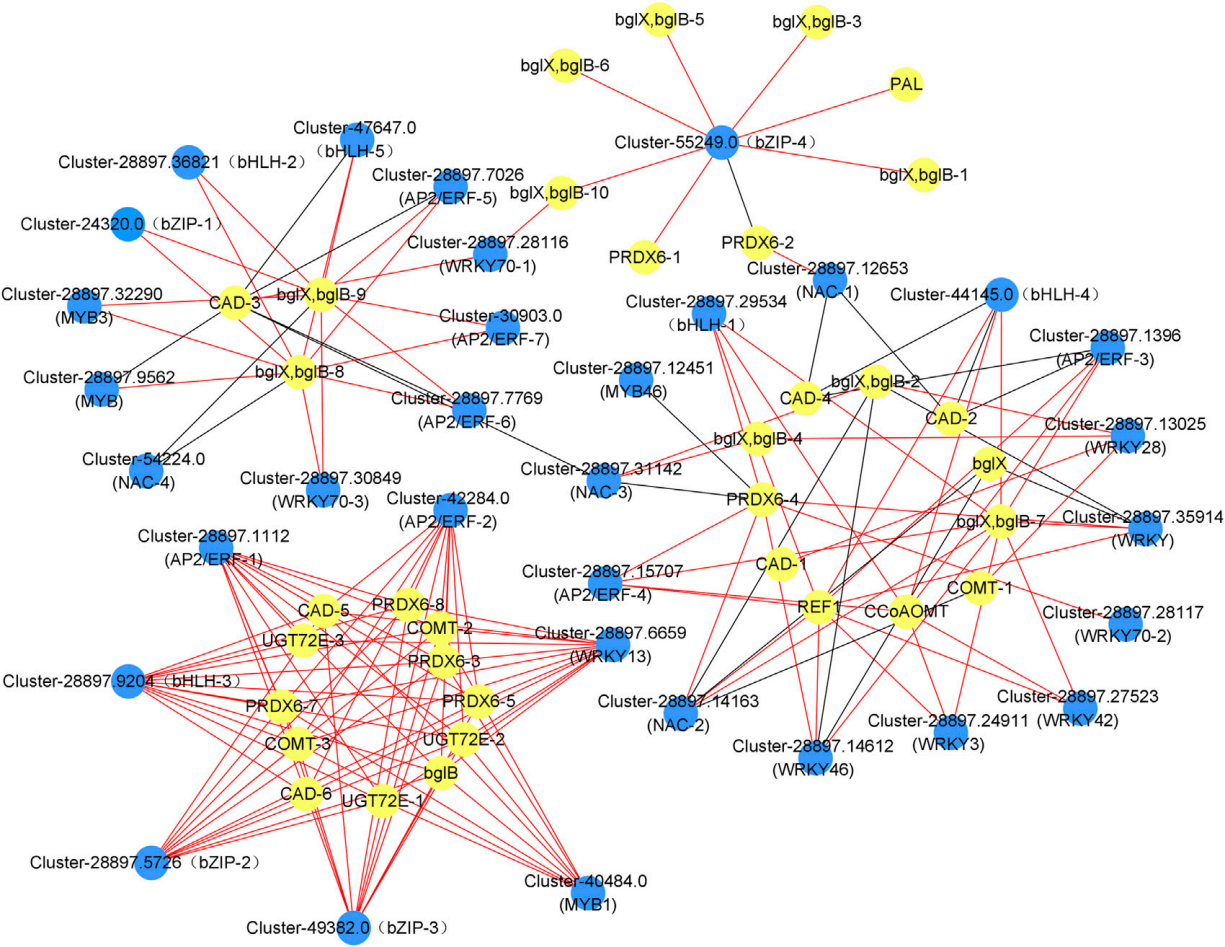
Co-expression analysis of the genes involved in the phenylpropanoid biosynthesis pathway and transcription factors (TFs) in the leaves and roots from white clover after dodder parasitism. Blue nodes indicate TFs. Yellow nodes indicate genes related to phenylpropanoid biosynthesis. Red and dark grey lines indicate positive corrections and negative corrections, respectively.

We searched the dodder parasitism-induced transcriptome for TFs among the DEGs. Consequently, we annotated 109 of the DEGs into 35 transcription factor families, including 9 WRKYs, 7 AP2/ERFs, 5 bHLHs, 5 bZIPs, 4 MYBs and 5 NACs. We analyzed the correlation between the expression levels of these transcription factors and genes involved in phenylpropanoid synthesis. Accordingly, 9 WRKYs, 7 AP2/ERFs, 5 bHLHs, 4 bZIPs, 4 MYBs, and 4 NACs showed a close relationship with the phenylpropanoid synthesis, with the correlation coefficients >0.9, and the expression levels of majority TFs showed a positive correlation with those of the phenylpropanoid synthesis genes (Figure 6, Supplementary Table S10). Additionally, 25 TFs were highly related to the lignin synthesis, including 8 WRKYs, 6 AP2/ERFs, 4 bHLHs, 3 bZIPs, 3 MYBs, and 3 NACs, thereby suggesting that these TFs are important in the white clover defense against dodder parasitism.

### Validation of DEGs *via* RT-qPCR

**FIGURE 7:**
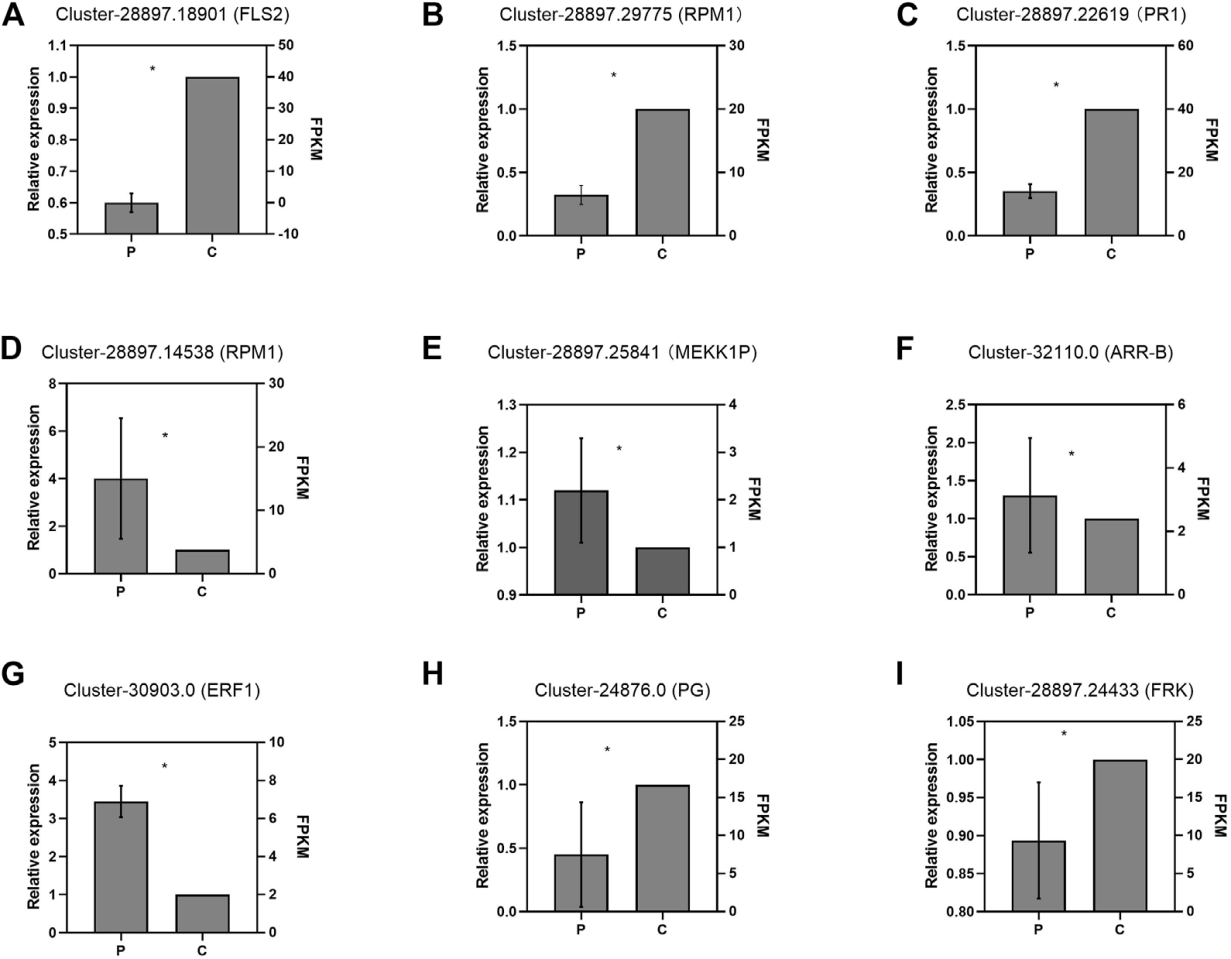
Validation of the expression profiles of partial DEGs by RT-qPCR: Panels **(A)**–**(C)** and **(D)**–**(I)** indicate the DEGs in the leaves and roots, respectively. P and C indicate the parasitized and non-parasitized hosts, respectively. Each value is calculated from three biological and technical replicates. The error bars represent the standard deviations (SD). Values with the different letters indicate significant differences according to the Independent-Samples *t*-Test (*p* < 0.05). Cluster-28897.18901 (*FLS2*): Plant-pathogen interaction/LRR receptor-like serine/threonine-protein kinase ERL1/LRR receptor-like serine/threonine-protein kinase FLS2; Cluster-28897.29775(*RPM1)*: Plant-pathogen interaction/disease resistance protein RPM1; Cluster-28897.22619(*PR1*): Plant hormone signal transduction/pathogenesis-related protein 1; Cluster-28897.14538(*RPM1)*: Plant-pathogen interaction/disease resistance protein RPM1; Cluster-28897.25841(*MEKK1P)*: Plant-pathogen interaction/mitogen-activated protein kinase kinase 1; Cluster-32110.0(*ARR-B)*: Plant hormone signal transduction/two-component response regulator ARR12-like protein; Cluster-30903.0 (*ERF1*): Plant hormone signal transduction/ethylene-responsive transcription factor 1b-like protein; Cluster-24876.0 (*PG):*Starch and sucrose metabolism/probable polygalacturonase; Cluster-28897.24433(*FRK):*Starch and sucrose metabolism/pfkB family carbohydrate kinase.

To validate the DEGs obtained by RNA-seq, we selected nine DEGs for real-time reverse transcription polymerase chain reaction (RT-qPCR) analysis, including four genes (Cluster-28897.18901-*FLS2*, Cluster-28897.29775-*RPM1*, Cluster-28897.145389-*RPM1,* and Cluster-28897.25841-*MEKK1P*) involved in plant-pathogen interaction, three genes (Cluster-28897.22619-*PR1*, Cluster-32110.0-*ARR-B,* and Cluster-30903.0-*ERF1*) involved in plant hormone signal transduction, and two genes (Cluster-24876.0-*PG* and Cluster-28897.24433-*FRK*) involved in starch and sucrose metabolism (Figure 7). The results showed that the expression patterns of these DEGs were consistent with those obtained from RNA-seq.

## Discussion

A previous report suggested that the host plants have a similar response mechanism against parasite plants and pathogen infection (Runyon et al., 2010a). Many genes are vital for recognizing PAMPs and subsequent activating anti-pathogen defense mechanisms, like R genes, protein kinase genes, and PR genes (Tariq et al., 2018). The physiological and biochemical impact of parasitism on hosts have been continually characterized and it has been established in quantitative terms that *Cuscuta* parasitism reduces the lushness of growth of hosts (Shen et al., 2007; Li et al., 2015). *Cuscuta* parasitism results in significant root biomass reduction and changes the biomass allocation patterns, resulting in drastic increase of shoot: root dry weight ratio in parasitized hosts (Shen et al., 2005). There is a trade-off between plant growth and defense (Ballhorn et al., 2014). Competition or competitive behaviors can also affect the plant at various organizational levels resulting in morphological responses (plant growth), biochemical responses (plant defense) and resource allocation (Fernandez et al., 2016). In this study, more R genes were downregulated in parasitized leaves while more R genes were upregulated in parasitized roots, suggesting a trade-off between growth and defense in different tissues of host.

Plant hormones are signaling compounds that regulate crucial aspects of growth, development and different biotic and abiotic stress responses (Smith et al., 2009; Wani et al., 2016; Chen LM. et al., 2021). In this study, the expression of 19 genes related to six hormones (Auxin, CTK, ABA, ET, JA and SA) metabolism or signaling were significantly affected by dodder parasitism (Figure 4). The induction of plant defense is regulated by complex signaling networks, in which the phytohormones JA and SA play vital roles. Generally, JA-mediated pathway is involved in the defense response against herbivores, while the SA-mediated pathway helps regulate defense against microbial pathogens (Smith et al., 2009). However, studies on plant response to parasitic plants suggested that the crucial interaction between JA and SA is necessary to mediate effective defense. In tomato, JA and SA were sequentially induced to mediate effective defense against the *C. pentagona* infection. In the current study, one gene related to SA was differentially expressed in dodder affected leaves, while two genes related to SA and one gene related to JA were differentially expressed in dodder affected roots. Furthermore, the ABA content also increased after dodder attack (Runyon et al., 2010b). In this study, one and two genes related to ABA were differentially expressed in dodder affected leaves and roots, respectively, thus suggesting the complex signaling pathways regulated by plant hormones against parasitic plant infection.

The phenylpropanoid pathway is crucial for land plants to survive severe challenges, including desiccation stress, temperature stress, UV radiation, pests and pathogen infections. The phenylpropanoid pathway produces lignin, flavonoids, and other metabolites (Yadav et al., 2020). It has been reported that plants defend root and stem parasitic plants through the accumulation of lignin at the infection sites on roots and stems of host plants, respectively. For example, lignification of endodermal and pericycle host cells prevent parasite intrusion into the root vascular cylinder at early infection stages (Perez et al., 2005). An increased deposition of lignin was accumulated in the resistant rice cultivar upon root parasitic *S. hermonthica* infection. And the increased deposition of lignin was accompanied by induction of the expression of corresponding enzyme-encoding genes in the phenylpropanoid pathway (Mutuku et al., 2019). The resistant tomato cultivar Heinz exhibited a lignification-based resistance against the stem parasitic field dodder (*C. campestris*) infection (Jhu et al., 2022). PAL is important for providing precursors for various downstream metabolites. Downregulation of *PAL* can significantly decrease the lignin content in both *Arabidopsis* and *Populus* (Rohde et al., 2004; Wang CT et al., 2018; Zhang et al., 2020). In this study, the *PAL* expression (Cluster-28897.11757) was significantly upregulated in P_leaf_ vs. C_leaf_, thereby suggesting that sufficient precursors were available for the downstream lignin biosynthesis. Additionally, two *CAD* genes (Cluster-28897.12905 and Cluster-28897.21230), one *COMT* (Cluster-28897.5861) and one *PRDX6* (Cluster-33784.0) were upregulated in P_leaf_ vs. C_leaf_ (Figure 5A), which are important in the lignin monolignol synthesis in *Arabidopsis* (Rohde et al., 2004) and *Populus* (Zhang et al., 2020). Various reports have shown that a mobile signal was produced in the herbivores damaged local leaves and traveled to systemic leaves to activate defenses (Grenn & Ryan, 1972). For example, above-ground herbivores attacked *Triadica sebifera* leaves induced above- and below-ground defensive responses (Xiao et al., 2019). In this study, dodder parasitism on host stem also resulting in many transcriptomic changes in host roots, suggested that systemic signal produced at the dodder infection sites and transmitted to the distal roots. In terms of lignin biosynthesis, four *CAD* genes (Cluster-28897.16469, Cluster-28897.21230, Cluster-51220.0 and Cluster-28897.21232), two *COMT* genes (Cluster-57653.0 and Cluster-51096.0), six *PRDX6* genes (Cluster-28897.5019, Cluster-28897.34522, Cluster-28897.21461, Cluster-28897.22464, Cluster-28897.10573, and Cluster-28897.5566) and three *UGT72E* genes (Cluster-28897.10004, Cluster-28897.3248 and Cluster-19366.0) showed differential expression patterns in P_root_ vs. C_root_. Interestingly, more DEGs involved in the lignin biosynthesis displayed downregulation in P_root_ vs. C_root_ (Figure 5B), which indicated that the parasitized host might allocate more resources to leaves than roots to meet the requirement of lignin biosynthesis in leaves.

**FIGURE 8:**
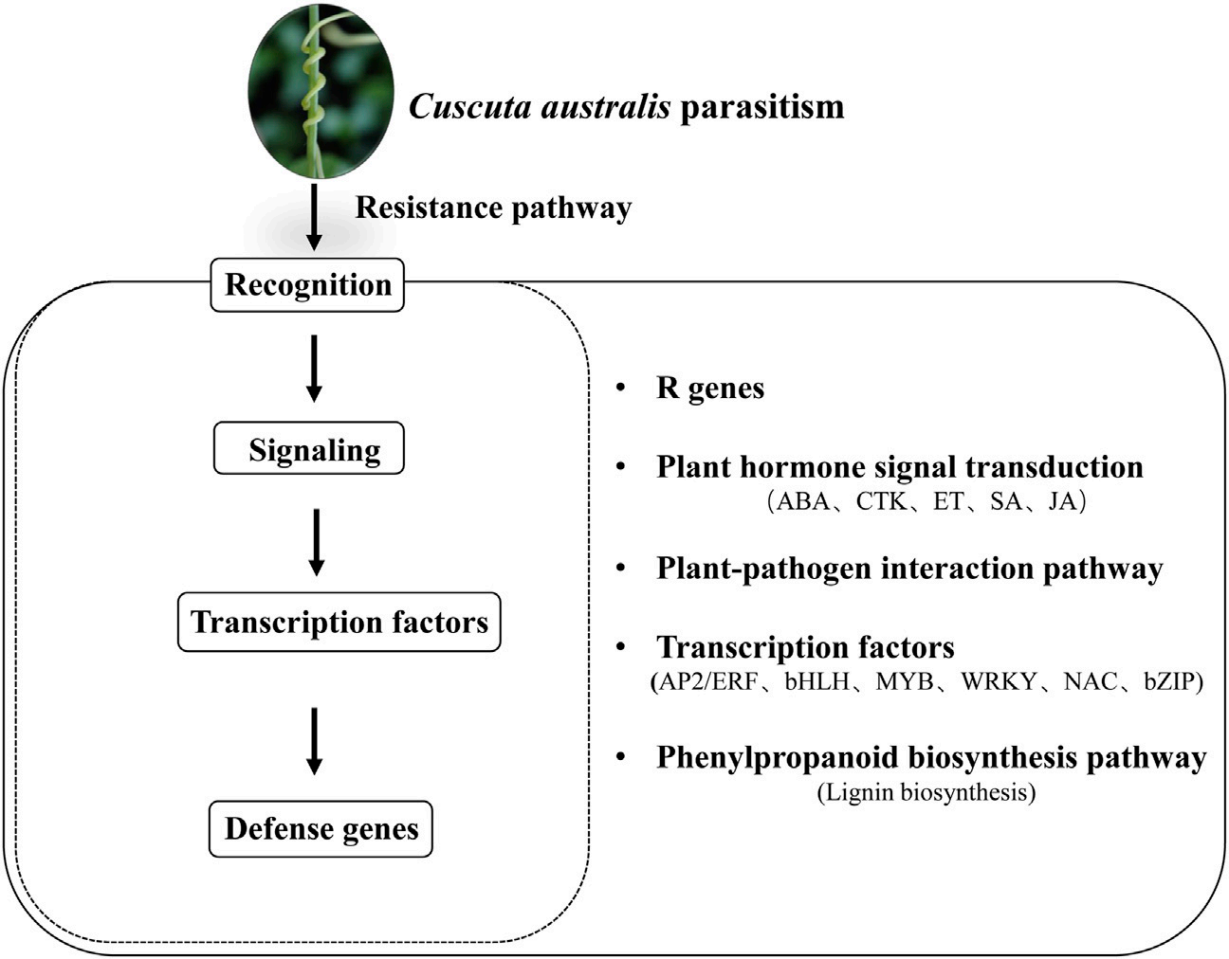
Schematic overview of response pathways of white clover to dodder parasitism.

In plants, transcription factors of the WRKY, AP2/ERF, bZIP, bHLH, MYB, and NAC families are important in gene regulatory networks against various biotic and abiotic stresses in different plants (Shen et al., 2012; Wang et al., 2018; Li et al., 2020; Yan et al., 2020; Gao et al., 2021; Herath & Verchot, 2021). Our study found that 9 WRKYs, 7 AP2/ERFs, 5 bHLHs, 4 bZIPs, 4 MYBs, and 4 NACs were closely correlated with genes involved in the phenylpropanoid synthesis and the lignin synthesis. Accumulation studies have shown that WRKYs are responsible for plant defense response to abiotic and biotic stresses (Wani et al., 2021). For example, in cotton, *GhWRKY41* directly activates the expression of *GhC4H* and *Gh4CL*, thereby contributing to the accumulation of flavonoids and lignin and enhancing the defense against *Verticillium dahlia* (Xiao et al., 2023). In a recent report, the resistant tomato cultivar Heinz showed a lignin-based resistance against field dodder parasitism (Jhu et al., 2022), which was regulated by a *CC-NBS-LRR* gene and three transcription factors, *SIWRKY16*, *SIMYB55,* and an *AP2* transcription factor *LIF1*. Specifically, *SIWRKY16* was upregulated in the Heinz tomato after the *C. campestris* parasitism (Jhu et al., 2022). AP2/ERF transcription factors have been reported to play an important role in lignin biosynthesis (Ma et al., 2005). For example, in *Eirobotrya japonica*, *EjAP2-1* was identified as a novel regulator of fruit lignification *via* interaction with *EjMYB* (Zeng et al., 2015); in rice, the overexpression of *Arabidopsis SHINE* (an AP2/ERF gene) resulted in a 45% reduction in lignin content compared with the wild type production (Ambavaram et al., 2011), suggesting that AP2/ERF had potential functions in lignin regulation. It is known that bZIP transcription factors can regulate plant defense against pathogen infection. For example, in potato, *StbZIP61* function together with *StNPR3L* to regulate the temporal activation of SA biosynthesis, resulting in SA-mediated immunity against *Phytophthora infestans* infection (Zhou et al., 2018). bHLH gene family has been reported to play vital roles in response to abiotic and biotic stresses. For example, the expression of *HabHLH024* was upregulated in both roots and leaves of a resistant sunflower cultivar under root parasite *Orobanche cumana* infection (Li et al., 2020). MYB transcription factors have been reported to play a key role in response to the infection of the root parasitic weed *O. cumana* on the sunflower (Li et al., 2020). Additionally, transcriptomic analyses of the parasitic plant *C. japonica* on both host and non-host plants have shown that the expression of *MYB* genes is upregulated in host plants compared to non-host plants. This upregulation of MYB genes is positively related to the genes involved in the phenylpropanoid biosynthesis pathway, which ultimately leads to stress responses in host plants (Guo et al., 2022). NAC transcription factors have been demonstrated to participate in plant-pathogen interaction as both positive or negative regulators of the downstream defense-related genes (Bian et al., 2020). NAC and MYB transcription factors are involved in the regulation of lignin biosynthesis (Chen L. et al., 2021). Recently, the co-expression network among MYB, NAC, and lignin biosynthesis genes was conducted in two rattan species, *Calamus simplicifolius*, and *D. jenkinsiana.* In *Daemonorops jenkinsiana,* four NACs were co-expressed with ten lignin biosynthesis genes, *C3H*, *CAD*, *CCoAOMT*, *CSE*, *F5H*, *C4H, CCR, PAL, LACCASES*, and *PEROXIDASES*, while 59 MYBs were co-expressed with 76 lignin biosynthesis genes. Among the co-expression relationships, *4CL, C4H, C3H, CCR, CAD, HCT, LACCASES*, and *PEROXIDASES* had binding sites for MYB transcription factors. This result suggests that these NACs and MYBs were profoundly involved in regulating the lignin biosynthesis pathway (Wang JP et al., 2018). Our result demonstrated that eight WRKYs, six AP2/ERFs, four bHLHs, three bZIPs, three MYBs, and three NACs were co-expressed with lignin biosynthesis genes. For example, *WRKY28* (Cluster-28897.13025) was co-expressed with *CAD-1* (Cluster-28897.12905) and *COMT-1* (Cluster-28897.5861); *MYB1* was co-expressed with *PRDX6-3/7* (Cluster-288978.5019/Cluster-28897.10573), *UGT72E-1/2* (Cluster-288978.10004/Cluster-28897.3248), *CAD-5* (Cluster-51220.0), and *COMT-2* (Cluster-57653.0); *NAC-1* (Cluster-28897.12653) was co-expressed with *PRDX6-2* (Cluster-28897.20416) and *CAD-2* (Cluster-28897.21230), suggesting that these TFs play a regulatory role in the lignin biosynthetic pathway and thus response against dodder parasitism (Figures 6, 8).

## Conclusion

A comparative transcriptomics of the leaves and roots from white clover both with and without dodder parasitism were conducted to explore the defense-related genes and pathways. We identified 1,329 and 3,271 DEGs in the leaf and roots samples, respectively. The KEGG pathway enrichment analysis revealed that the DEGs were mainly involved in ribosome, plant-pathogen interaction, plant hormone signal transduction, and phenylpropanoid biosynthesis. The results showed that 8 *WRKY*s, 6 *AP2/ERF*s, 4 *bHLH*s, 3 *bZIP*s, 3 *MYB*s and 3 *NAC*s were closely correlated with lignin biosynthesis, which may play vital roles in the white clover response against dodder parasitism. Thus, our results provide new insights into understanding the complex regulatory network behind the parasite-host plant interactions.

## Data Availability

The datasets presented in this study can be found in online repositories. The names of the repository/repositories and accession number (s) can be found in the article/Supplementary Material.
