# Supplementary material for: Transcriptome analysis reveals defense-related genes and pathways during dodder (Cuscuta australis) parasitism on white clover (Trifolium repens)
Source: Front Genet. 2023 Mar 16;14:1106936. doi: 10.3389/fgene.2023.1106936 (PMC10060986; doi:10.3389/fgene.2023.1106936)
Supplement: Supplementary file 1 [file DataSheet1.PDF]

Supplementary Material

1 Supplementary Figures and Tables

1.1 Supplementary Figures

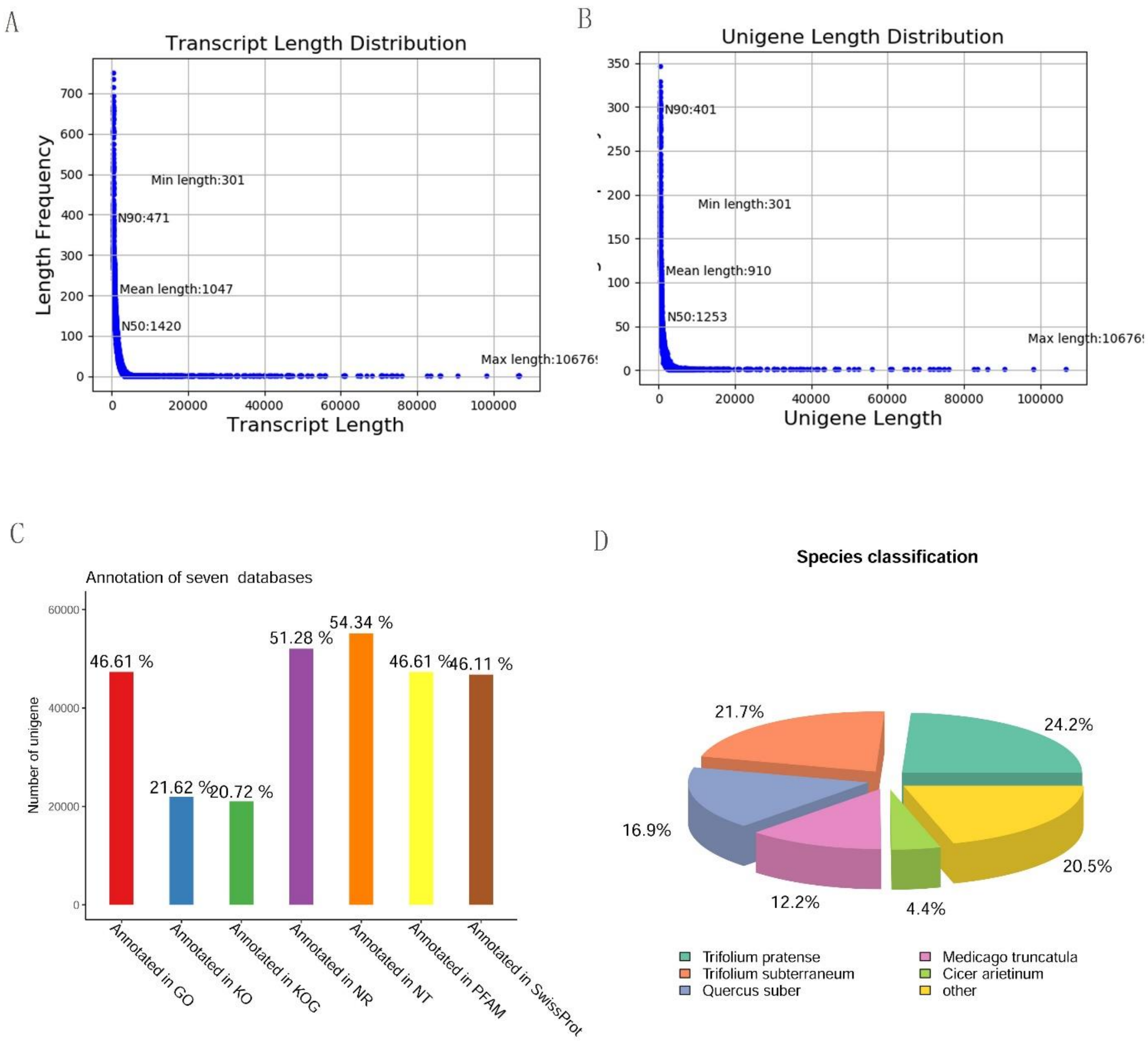

Supplementary Figure 1. Analysis of the transcriptome data (A): Length distribution of the transcripts; (B): Length distribution of the unigenes; (C): Transcriptome data annotated in the seven databases; (D): The species classification of the transcriptome data annotated using the NR database.

(A)

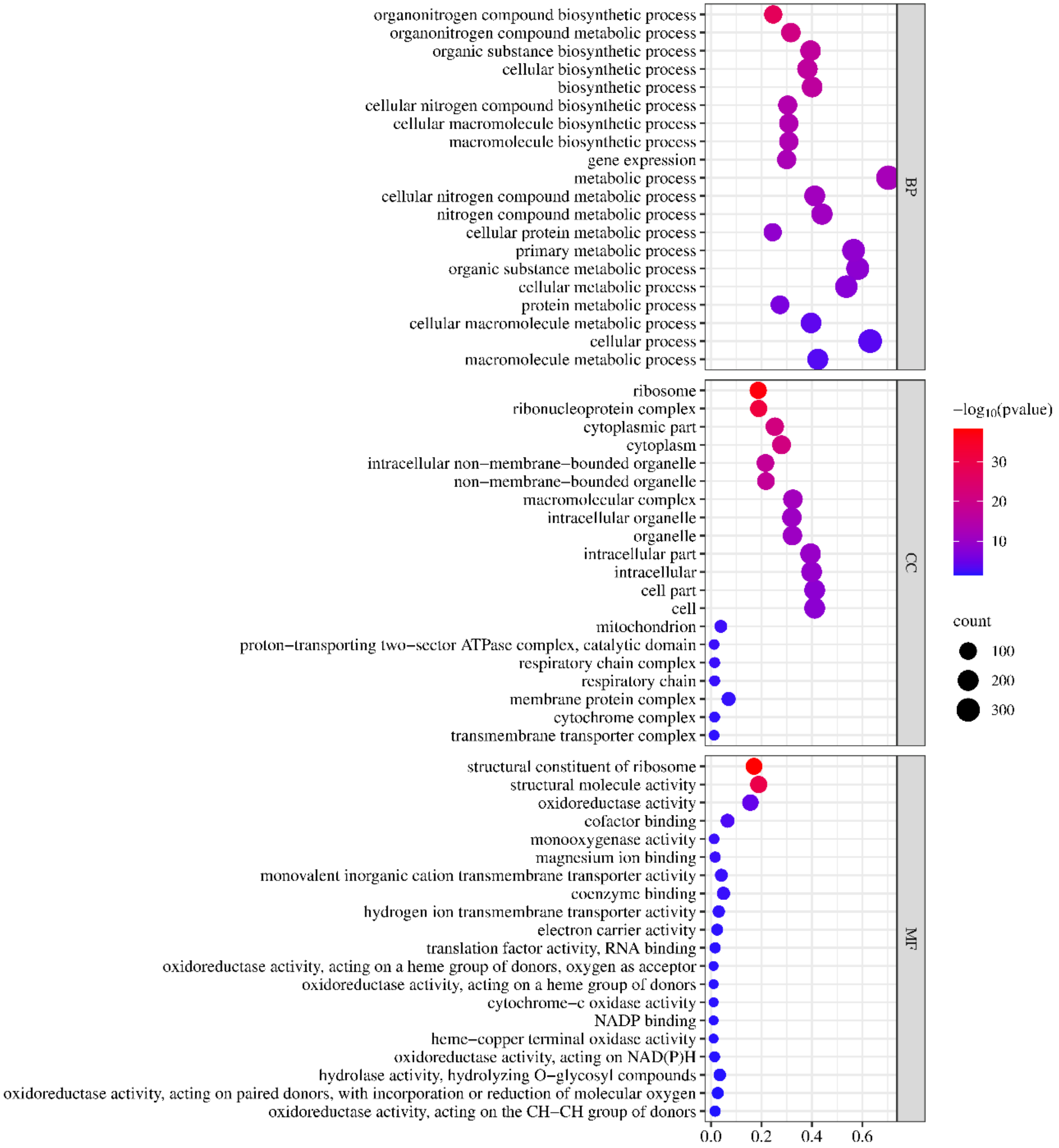
organonitrogen compound biosynthetic process
organonitrogen compound metabolic process
organic substance biosynthetic process
cellular biosynthetic process
biosynthetic process
cellular nitrogen compound biosynthetic process
cellular macromolecule biosynthetic process
macromolecule biosynthetic process
gene expression
metabolic process
cellular nitrogen compound metabolic process
nitrogen compound metabolic process
cellular protein metabolic process
primary metabolic process
organic substance metabolic process
cellular metabolic process
protein metabolic process
cellular macromolecule metabolic process
cellular process
macromolecule metabolic process
BP
ribosome
ribonucleoprotein complex
cytoplasmic part
cytoplasm
intracellular non−membrane−bounded organelle
non−membrane−bounded organelle
macromolecular complex
intracellular organelle
organelle
intracellular part
intracellular
cell part
cell
mitochondrion
proton−transporting two−sector ATPase complex, catalytic domain
respiratory chain complex
respiratory chain
membrane protein complex
cytochrome complex
transmembrane transporter complex
CC
structural constituent of ribosome
structural molecule activity
oxidoreductase activity
cofactor binding
monooxygenase activity
magnesium ion binding
monovalent inorganic cation transmembrane transporter activity
coenzyme binding
hydrogen ion transmembrane transporter activity
electron carrier activity
translation factor activity, RNA binding
oxidoreductase activity, acting on a heme group of donors, oxygen as acceptor
oxidoreductase activity, acting on a heme group of donors
cytochrome−c oxidase activity
NADP binding
heme−copper terminal oxidase activity
oxidoreductase activity, acting on NAD(P)H
hydrolase activity, hydrolyzing O−glycosyl compounds
oxidoreductase activity, acting on paired donors, with incorporation or reduction of molecular oxygen
oxidoreductase activity, acting on the CH−CH group of donors
MF
0.0
0.2
0.4
0.6
−log10(pvalue)
30
20
10
count
100
200
300

(B)

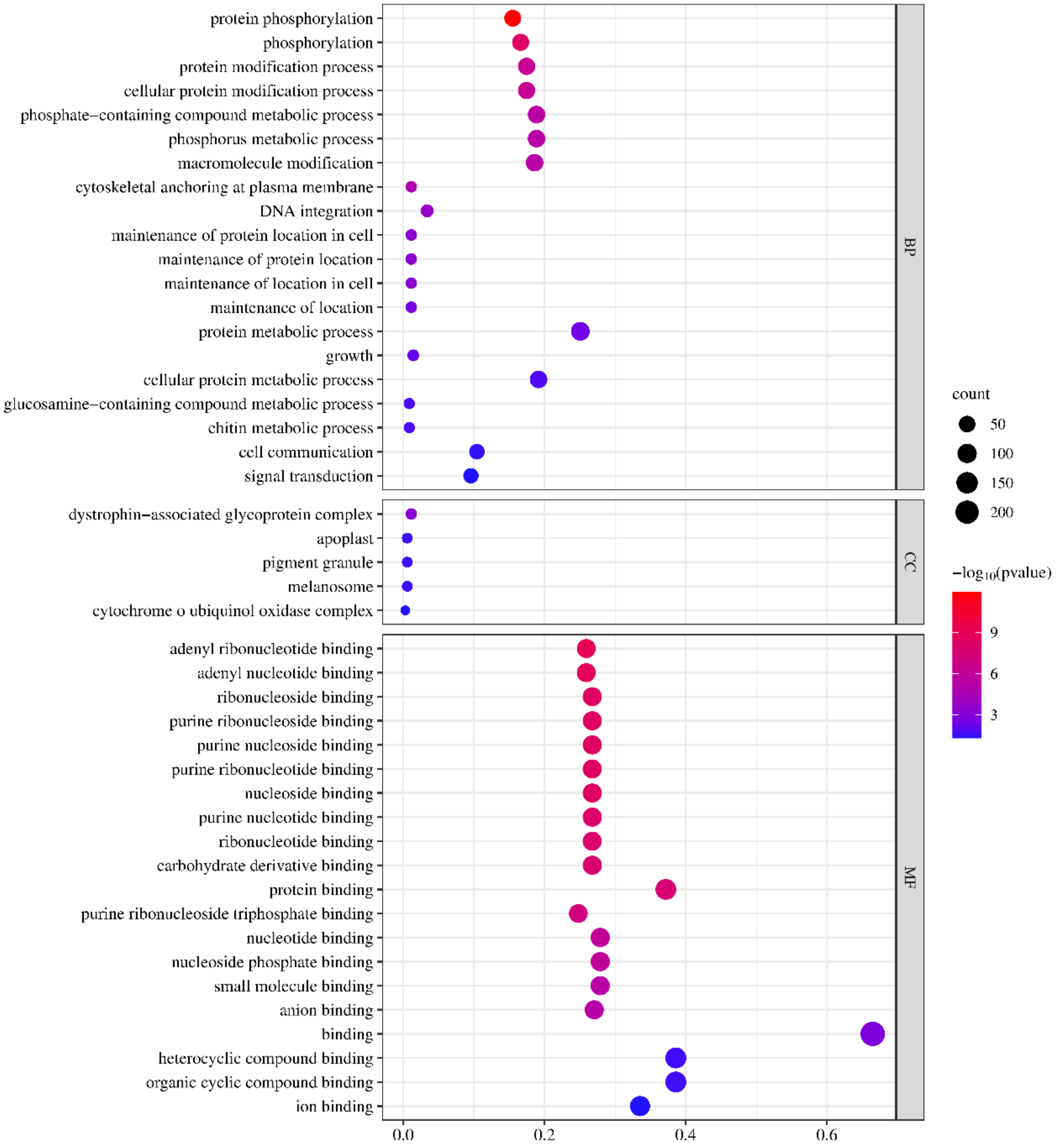
protein phosphorylation
phosphorylation
protein modification process
cellular protein modification process
phosphate−containing compound metabolic process
phosphorus metabolic process
macromolecule modification
cytoskeletal anchoring at plasma membrane
DNA integration
maintenance of protein location in cell
maintenance of protein location
maintenance of location in cell
maintenance of location
protein metabolic process
growth
cellular protein metabolic process
glucosamine−containing compound metabolic process
chitin metabolic process
cell communication
signal transduction
dystrophin−associated glycoprotein complex
apoplast
pigment granule
melanosome
cytochrome o ubiquinol oxidase complex
adenyl ribonucleotide binding
adenyl nucleotide binding
ribonucleoside binding
purine ribonucleoside binding
purine nucleoside binding
purine ribonucleotide binding
nucleoside binding
purine nucleotide binding
ribonucleotide binding
carbohydrate derivative binding
protein binding
purine ribonucleoside triphosphate binding
nucleotide binding
nucleoside phosphate binding
small molecule binding
anion binding
binding
heterocyclic compound binding
organic cyclic compound binding
ion binding
0.0
0.2
0.4
0.6
BP
CC
MF
count
50
100
150
200
−log10(pvalue)
9
6
3

(C)

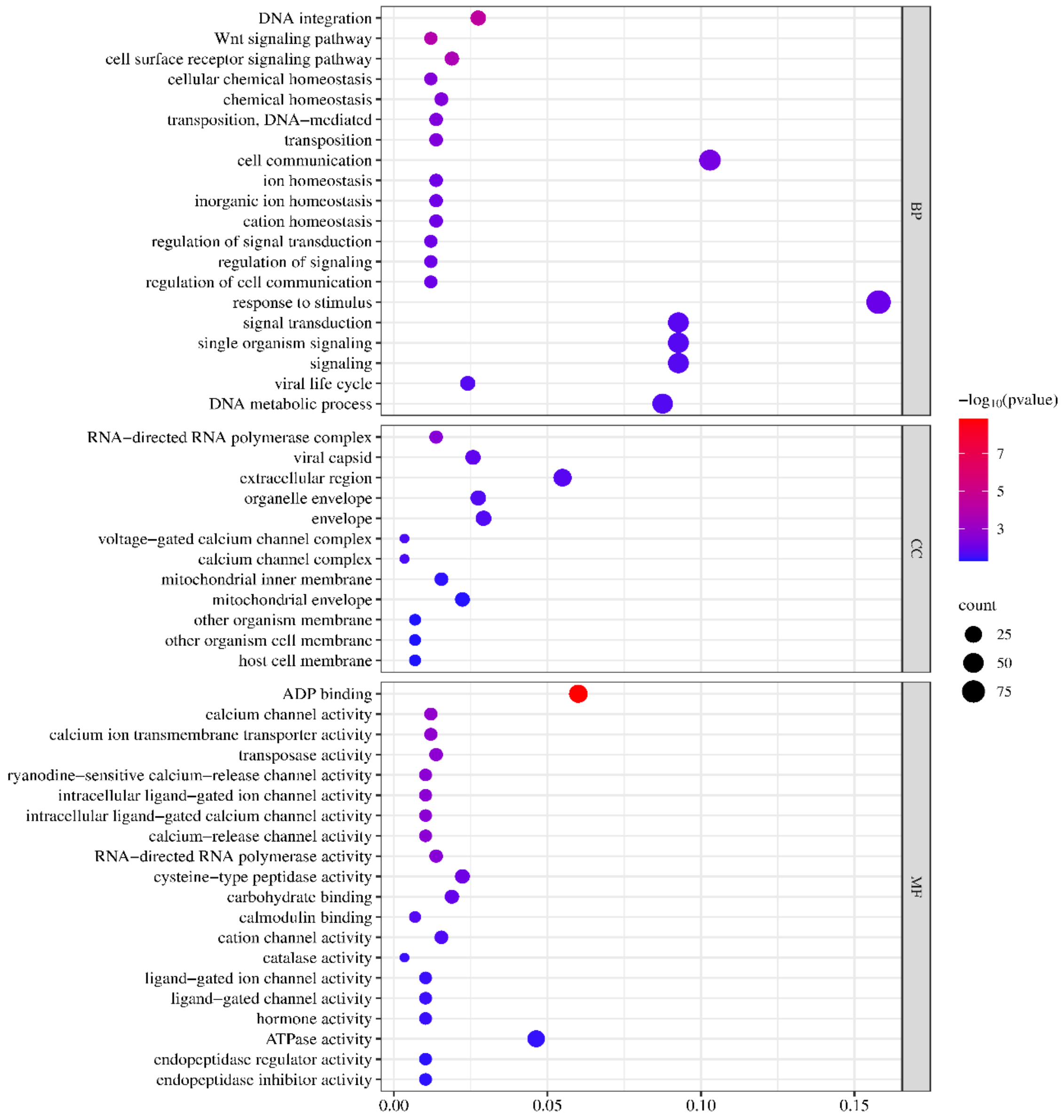
DNA integration
Wnt signaling pathway
cell surface receptor signaling pathway
cellular chemical homeostasis
chemical homeostasis
transposition, DNA−mediated
transposition
cell communication
ion homeostasis
inorganic ion homeostasis
cation homeostasis
regulation of signal transduction
regulation of signaling
regulation of cell communication
response to stimulus
signal transduction
single organism signaling
signaling
viral life cycle
DNA metabolic process
BP
RNA−directed RNA polymerase complex
viral capsid
extracellular region
organelle envelope
envelope
voltage−gated calcium channel complex
calcium channel complex
mitochondrial inner membrane
mitochondrial envelope
other organism membrane
other organism cell membrane
host cell membrane
CC
ADP binding
calcium channel activity
calcium ion transmembrane transporter activity
transposase activity
ryanodine−sensitive calcium−release channel activity
intracellular ligand−gated ion channel activity
intracellular ligand−gated calcium channel activity
calcium−release channel activity
RNA−directed RNA polymerase activity
cysteine−type peptidase activity
carbohydrate binding
calmodulin binding
cation channel activity
catalase activity
ligand−gated ion channel activity
ligand−gated channel activity
hormone activity
ATPase activity
endopeptidase regulator activity
endopeptidase inhibitor activity
MF
0.00
0.05
0.10
0.15
−log10(pvalue)
7
5
3
count
25
50
75

(D)

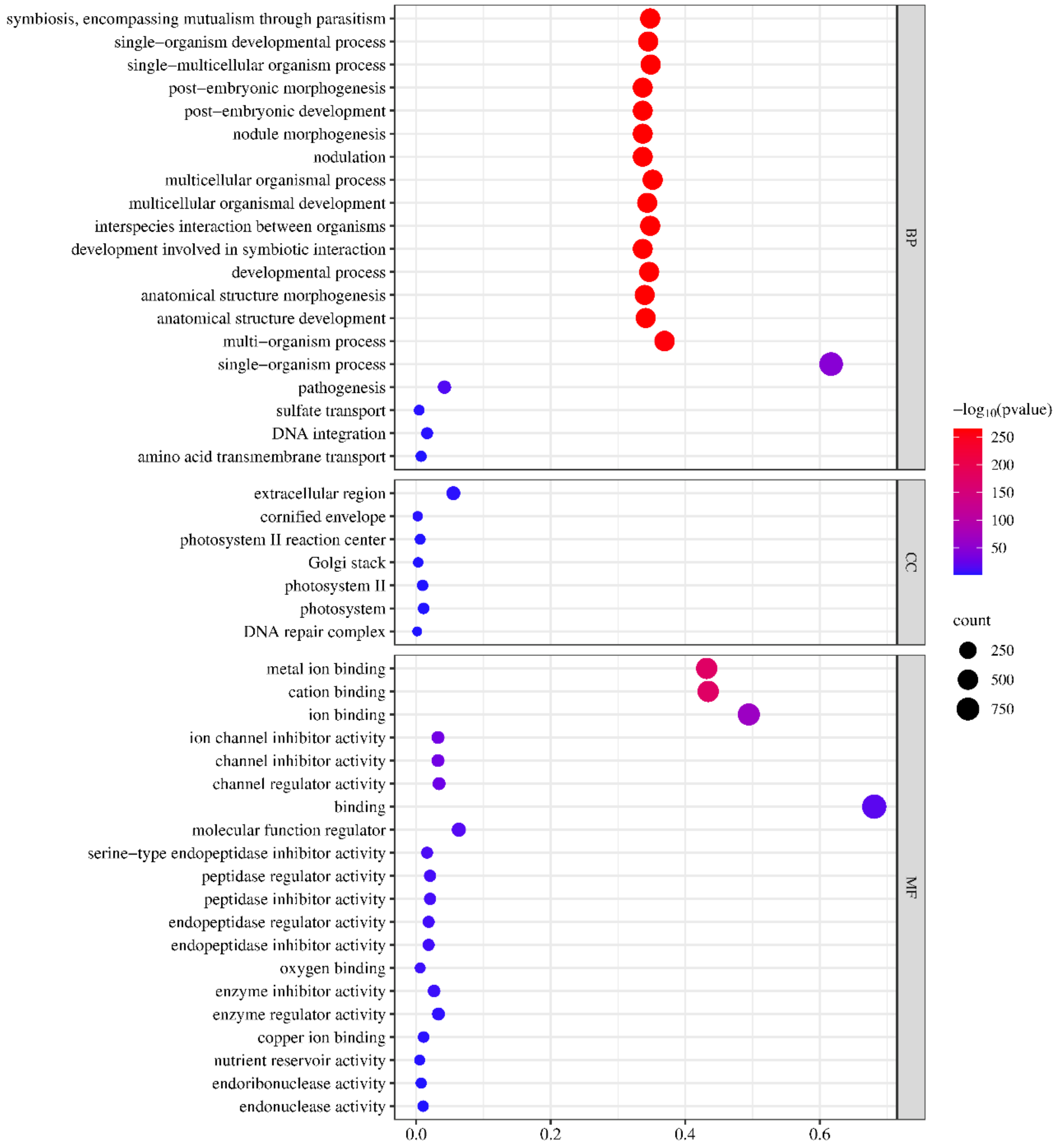
symbiosis, encompassing mutualism through parasitism
single−organism developmental process
single−multicellular organism process
post−embryonic morphogenesis
post−embryonic development
nodule morphogenesis
nodulation
multicellular organismal process
multicellular organismal development
interspecies interaction between organisms
development involved in symbiotic interaction
developmental process
anatomical structure morphogenesis
anatomical structure development
multi−organism process
single−organism process
pathogenesis
sulfate transport
DNA integration
amino acid transmembrane transport
BP
extracellular region
cornified envelope
photosystem II reaction center
Golgi stack
photosystem II
photosystem
DNA repair complex
CC
metal ion binding
cation binding
ion binding
ion channel inhibitor activity
channel inhibitor activity
channel regulator activity
binding
molecular function regulator
serine−type endopeptidase inhibitor activity
peptidase regulator activity
peptidase inhibitor activity
endopeptidase regulator activity
endopeptidase inhibitor activity
oxygen binding
enzyme inhibitor activity
enzyme regulator activity
copper ion binding
nutrient reservoir activity
endoribonuclease activity
endonuclease activity
MF
0.0
0.2
0.4
0.6
−log10(pvalue)
250
200
150
100
50
count
250
500
750

Supplementary Figure 2. GO classification for DEGs in white clover with dodder parasitism.

(A): up-regulated DEGs in Pleaf vs Cleaf; (B): down-regulated DEGs in Pleaf vs Cleaf; (C): up-regulated DEGs in Proot vs Croot; (D): down-regulated DEGs in Proot vs Croot. The Rich factor is the ratio of number of differentially expressed genes annotated in this pathway to the number of all genes annotated in this pathway. The higher the Rich factor, the greater the degree of pathway enrichment. A q-value is an adjusted p value; taking into account the false discovery rate and range from 0 to 1 and a lower value indicates greater pathway enrichment.

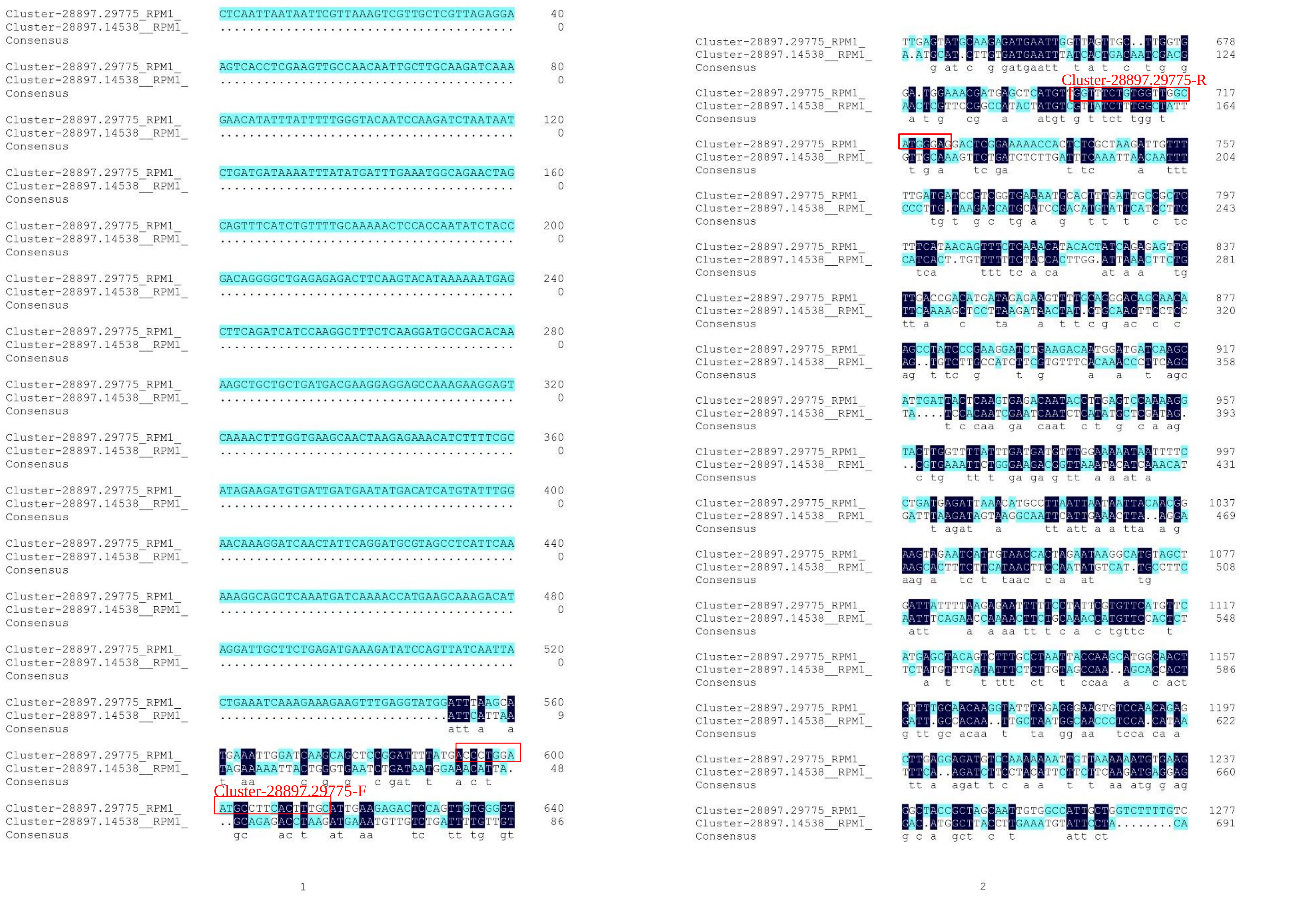
Cluster-28897.29775-R
Cluster-28897.29775-F
1
2

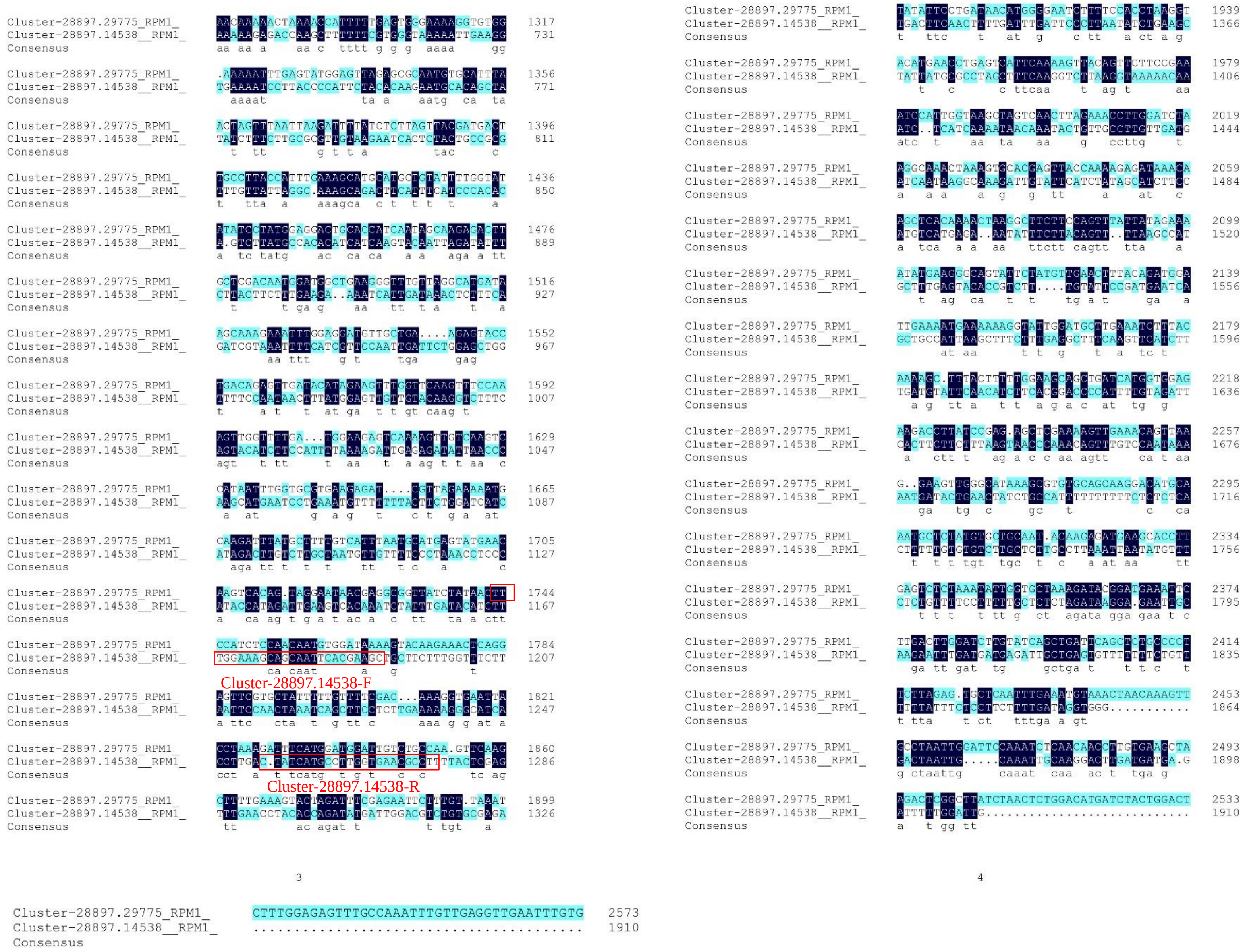
Cluster-28897.29775_RPM1_
Cluster-28897.14538__RPM1_
Consensus
Cluster-28897.14538-F
Cluster-28897.14538-R
3
4

Supplementary Figure 3. Sequence alignment of two RPM1 genes Cluster-28897.29775 and Cluster-28897.14538. The primer pairs were highlight in the sequences.

1.2 Supplementary Tables

Supplementary Table S1. Primers used in the RT-qPCR validation of RNA-Seq in this study

Gene ID and name	KEGG category/gene annotation	Forward 5'-3'	Reverse 5'-3'	RNA Source Tissue
Cluster-28897.18901 （FLS2）	Plant-pathogen interaction/ LRR receptor-like serine/threonine-protein kinase ERL1/ LRR receptor-like serine/threonine-protein kinase FLS2	AGTCCAAGGAAATCAAGCCTACCA	TGCAGTTTCTCTCCTTAGCGCA	Leaf
Cluster-28897.29775 （RPM1）	Plant-pathogen interaction/ disease resistance protein RPM1	ACCCTGGAATGCCTTCACTTTGC	CTCCCATGCCAACCACAGAAACC	Leaf
Cluster-28897.22619 （PR1)	Plant hormone signal transduction/ pathogenesis-related protein 1	CAGCTACGGTGTCGTCCCAAAC	TGCACAAGACTCGCCAACAGAC	Leaf
Cluster-28897.14538 （RPM1）	Plant-pathogen interaction/ disease resistance protein RPM1	TTGGAAAGCAGCAATTCACGAAGC	AAGGCGTTCACCAAGGCATGATAG	Root

Cluster-28897.25841（MEKK1P）	Plant-pathogen interaction/ mitogen-activated protein kinase kinase kinase 1	AACGAAGCACTACTACCCAACAGC	CGCAAGCATCTGCCTATACGAGAC	Root
Cluster-32110.0（ARR-B）	Plant hormone signal transduction/ two-component response regulator ARR12-like protein	CAAGGGAGCATGTGTCTAGCCATC	TGAGAGGACTGAGGAGCCACTTAG	Root
Cluster-30903.0（ERF1）	Plant hormone signal transduction/ ethylene-responsive transcription factor 1b-like protein	GCCGCGTCCTCACTCCTCTATAG	ACCACCACCACCGATATCAATTGC	Root
Cluster-24876.0（PG）	Starch and sucrose metabolism/ probable polygalacturonase	TACCCTTGTCCTCCCATCCATGTC	CATAAGTGTGGGAAGCCTCGGAAG	Root
Cluster-28897.24433（FRK）	Starch and sucrose metabolism/ pfkB family carbohydrate kinase	ATCCCTGCTCATGCTTTGCTGAC	CCCCGGCCTAGAGCGTGATC	Root
EF1	Elongation Factor 1	TGATCCTGCACGGTTCACTTCTTC	GTTGGAGTCATCAAGGCTGTGGAG	Root and Leaf

Supplementary Table S2. Statistics of the white clover transcriptome sequencing data

Sample	Raw reads	Clean reads	Clean	Error	Q20	Q30	GC
P_{leaf1}	298,829,222	26,894,630	3.84	0.01	99.33	97.8	42.63
P_{leaf2}	343,225,466	30,890,292	4.45	0.01	99.37	97.88	43.46
P_{leaf3}	370,887,288	33,379,856	4.81	0.01	99.32	97.74	41.85
C_{leaf1}	359,716,711	32,374,504	4.67	0.01	99.35	97.84	42.54
C_{leaf2}	352,135,288	31,692,176	4.56	0.01	99.4	98	42.08
C_{leaf3}	371,253,555	33,412,820	4.81	0.01	99.39	97.95	41.88
P_{root1}	361,584,733	32,542,626	4.67	0.01	99.36	97.88	42.07
P_{root2}	374,996,777	33,749,710	4.86	0.01	99.35	97.86	42.05
P_{root3}	357,872,711	32,208,544	4.63	0.01	99.37	97.93	42.08
C_{root1}	372,680,844	33,541,276	4.83	0.01	99.36	97.9	41.41
C_{root2}	385,708,466	34,713,762	4.98	0.01	99.32	97.77	42.17
C_{root3}	382,977,711	34,467,994	4.95	0.01	99.36	97.91	41.42
Summary	3,948,891,061	355,400,196	51.11				

Supplementary Table S3. Identification of resistance genes in up-regulated DEGs from Pleaf vs Cleaf

gene_id	Pleaf_readcount	Cleaf_readcount	log2FoldChange	pval	padj	Gene length
**disease resistance protein**						
Cluster-28897.31079	93.52505676	6.157376	3.9138	0.00069	0.0447	350
Cluster-36079.0	320.7318388	0.561479	9.1134	3.93E-15	3.60E-11	1163
**pathogenesis**						
Cluster-28897.10823	125.7968404	3.260465	5.2373	4.47E-08	2.86E-05	738
Cluster-28897.10394	53.20235119	2.69953	4.3166	1.77E-06	0.000567	689
Cluster-50963.0	329.4994357	3.362025	6.6266	2.92E-06	0.000813	1369
Cluster-28897.4073	25.81815233	0	7.3246	1.13E-05	0.002143	848
Cluster-28897.10392	50.54734149	3.998516	3.6451	3.96E-05	0.005675	371
Cluster-9042.0	56.14338595	0	8.4486	4.83E-05	0.006594	704
Cluster-26585.0	41.91520922	0	8.0264	0.000226	0.020299	967
**protein kinase**						
Cluster-28897.8053	179.8170702	46.01851	1.9637	3.75E-06	0.000974	1055
Cluster-28897.34756	305.3693901	73.65167	2.0537	4.77E-06	0.001158	1740
Cluster-28897.10916	397.3639122	118.9088	1.7411	5.13E-05	0.006916	1438
Cluster-28897.35682	459.6632071	80.43649	2.5146	0.000118	0.012659	3438
Cluster-28897.17508	873.9304892	432.7808	1.0142	0.000265	0.022818	2084
Cluster-28897.24656	2832.039166	1000.225	1.5013	0.00078	0.048477	2590
**ubiquitin**						
Cluster-28897.33771	320.490265	0.629644	9.0514	7.42E-08	4.40E-05	702
Cluster-43998.0	296.8145879	1.242862	7.9306	6.86E-07	0.000263	801

Cluster-28897.34662	135.7371371	23.05839	2.559	2.20E-06	0.000665	722
Cluster-24193.1	108.9434598	0.298397	8.4429	6.01E-06	0.001359	1030
Cluster-31249.0	74.13009961	0	8.8491	1.79E-05	0.003069	452
Cluster-28897.10073	504.4097356	15.75971	5.0032	5.25E-05	0.007036	819
Cluster-28897.34083	155.5306064	4.585998	5.0949	5.91E-05	0.00774	490
Cluster-36644.0	22.21196625	0.263082	6.1456	0.000135	0.014058	514
Cluster-9374.0	45.0209556	0	8.1298	0.000154	0.015477	415
Cluster-28897.34092	55.10691085	0.577904	6.5721	0.000178	0.017196	565
Cluster-28897.2917	34.80095323	4.278023	3.0136	0.000195	0.018409	1487
Cluster-9651.0	37.84041635	0	7.8792	0.000249	0.021685	1011
Cluster-20041.1	38.03255925	0	7.8865	0.000285	0.024029	932
Cluster-55571.0	61.5796986	0.629644	6.6629	0.000343	0.027561	896
Cluster-8336.0	27.47024336	0	7.4117	0.000514	0.037077	400
Cluster-46139.0	30.53011017	3.122215	3.2715	0.000777	0.048408	1139
Cluster-28897.3924	19.83732773	0.613218	5.0613	0.00078	0.048477	314
**calcium**						
Cluster-28897.34662	135.7371371	23.05839	2.559	2.20E-06	0.000665	722
Cluster-28897.35648	233.2709123	36.60888	2.6723	4.83E-06	0.001166	1513
Cluster-28897.27133	375.4600947	103.922	1.851	6.90E-05	0.008725	1028
Cluster-42053.0	127.3056705	1.733712	6.1973	0.000117	0.012614	2141
Cluster-36644.0	22.21196625	0.263082	6.1456	0.000135	0.014058	514
Cluster-9069.0	40.31319413	0	7.9704	0.000211	0.019493	897
Cluster-26585.0	41.91520922	0	8.0264	0.000226	0.020299	967
Cluster-12534.1	85.37281952	1.207548	6.161	0.000237	0.020993	1403
Cluster-8612.0	38.87163355	0	7.9175	0.000249	0.021685	618

Cluster-29188.0	28.21623102	0	7.4553	0.000475	0.034948	385

Supplementary Table S4. Identification of resistance genes in down-regulated DEGs from Pleaf vs Cleaf

gene_id	Pleaf_readcount	Cleaf_readcount	log2FoldChange	pval	padj	Gene Length
**disease resistance protein**						
Cluster-28897.28712	13.27594	152.4068	-3.5057	3.34E-09	3.02E-06	1764
Cluster-28897.28675	2.5598	102.2499	-5.2451	3.31E-08	2.25E-05	1286
Cluster-28897.26723	175.6009	588.5911	-1.744	1.15E-07	6.13E-05	1688
Cluster-28897.19245	54.00942	381.4971	-2.8254	1.77E-07	8.81E-05	639
Cluster-28897.28265	0	44.08877	-7.7866	3.45E-07	0.00015	606
Cluster-28897.29775	139.907	516.8359	-1.8843	4.73E-07	0.00019	2573
Cluster-28897.28705	10.99395	80.86194	-2.8662	6.26E-07	0.000244	335
Cluster-28897.19635	2.004171	57.74522	-4.7937	1.09E-06	0.000383	1314
Cluster-28897.2637	0.833444	50.54784	-5.6761	1.86E-06	0.000581	1713
Cluster-28897.8143	0	30.60235	-7.2606	2.66E-06	0.000761	949
Cluster-28897.28678	30.10813	208.2981	-2.7747	5.65E-06	0.001308	790
Cluster-48181.0	13.95321	134.9785	-3.261	7.13E-06	0.001532	3277
Cluster-28897.15652	0.917765	36.36032	-5.158	1.10E-05	0.002104	570
Cluster-28897.22037	9.777575	106.665	-3.4221	1.47E-05	0.002643	1487
Cluster-28897.15963	11.58285	94.10802	-3.0379	2.50E-05	0.003954	644
Cluster-28897.7908	10.18266	70.41916	-2.8041	3.29E-05	0.004824	1159
Cluster-28897.19241	12.4244	148.4083	-3.5497	4.21E-05	0.005956	608

Cluster-28897.18529	10.30835	61.8015	-2.5541	5.00E-05	0.006799	874
Cluster-28897.28679	10.34837	140.2694	-3.7302	5.02E-05	0.006808	919
Cluster-28897.15388	42.71784	208.4052	-2.2759	5.49E-05	0.007314	945
Cluster-28897.36164	35.58047	254.0233	-2.8365	5.61E-05	0.007429	1117
Cluster-28897.26714	3.162431	98.18372	-4.918	6.82E-05	0.008655	1047
Cluster-28897.28690	59.49702	378.0427	-2.6743	7.94E-05	0.009496	1230
Cluster-28897.19251	27.12403	139.959	-2.3525	8.59E-05	0.010083	796
Cluster-28897.17160	9.389237	64.66254	-2.7749	9.74E-05	0.011161	1328
Cluster-28897.29970	21.04822	115.8211	-2.4781	0.000159	0.015844	363
Cluster-28897.25353	4.780965	62.39395	-3.663	0.000169	0.016514	558
Cluster-28897.16199	12.68974	148.8652	-3.5255	0.000208	0.019255	621
Cluster-28897.19246	83.88525	378.1574	-2.1712	0.000224	0.020235	1437
Cluster-28897.11641	4.080195	66.13755	-3.9745	0.000236	0.020927	2858
Cluster-28897.25351	8.34015	100.2859	-3.5806	0.000271	0.023205	917
Cluster-28897.28479	19.99778	163.8835	-3.0494	0.000294	0.024637	1489
Cluster-28897.13093	1.751208	32.68182	-4.0858	0.0003	0.025008	1991
Cluster-28897.21816	157.3133	381.0541	-1.2764	0.000411	0.031308	1912
Cluster-28897.9251	0.63995	25.97254	-5.2362	0.000432	0.032427	503
Cluster-28897.5520	1.687687	43.02443	-4.7822	0.000474	0.034927	1752
Cluster-28897.14592	23.22756	107.8613	-2.1919	0.000565	0.039472	1079
Cluster-28897.16010	2.776794	32.69578	-3.478	0.00061	0.041423	452
Cluster-28897.28711	1.303401	23.88542	-4.137	0.000647	0.043005	370
Cluster-28897.17070	13.70565	122.283	-3.1303	0.000706	0.045173	1410
Cluster-28897.28652	7.038099	55.43773	-2.9541	0.000738	0.046655	2630

Cluster-43557.0	0	43.27381	-7.7608	9.28E-05	0.010721	1704
Cluster-28897.12273	214.2313	1066.868	-2.3156	0.000672	0.043972	2027
Cluster-28897.15650	138.365	932.5883	-2.7547	7.89E-08	4.55E-05	1856
Cluster-28897.16178	41.18273	323.518	-2.9824	4.29E-07	0.000176	1259
Cluster-43885.0	0.362135	184.3685	-8.8897	1.92E-06	0.00059	2193
Cluster-43557.0	0	43.27381	-7.7608	9.28E-05	0.010721	1704
Cluster-28897.12979	155.1388	647.6034	-2.0659	9.78E-05	0.011173	1797
Cluster-28897.14979	0.917765	30.13204	-4.9078	0.000216	0.019731	1420
**pathogenesis**						
Cluster-28897.22619	17.80831	202.1314	-3.516	1.84E-06	0.000581	750
Cluster-28897.33168	1.665536	37.01624	-4.4263	1.86E-05	0.003169	1055
Cluster-51331.0	0.832093	34.20692	-5.5135	8.28E-05	0.009818	797
Cluster-52428.0	7.984996	99.27355	-3.6581	0.000104	0.011621	4569
Cluster-28897.16419	858.2107	2583.009	-1.5895	0.00017	0.016603	651
Cluster-28897.14551	337.8188	765.0629	-1.1806	0.000243	0.021395	2745
**protein kinase**						
Cluster-28897.11596	3.16108	97.95599	-4.9562	5.49E-06	0.001283	1343
Cluster-28897.12030	0	35.48458	-7.4756	2.86E-05	0.004346	565
Cluster-28897.12775	3.308923	90.76273	-4.7068	3.35E-05	0.004899	1709
Cluster-28897.13031	0	18.64677	-6.5447	0.000595	0.040709	341
Cluster-28897.13100	7.391819	244.8803	-5.0056	2.07E-05	0.003404	3214
Cluster-28897.13532	19.50139	101.3349	-2.3753	2.62E-05	0.0041	1380
Cluster-28897.13890	21.76686	131.3638	-2.5984	0.000437	0.032731	1818
Cluster-28897.15305	9.371139	119.6053	-3.6352	0.000142	0.014558	1568

Cluster-28897.15306	54.21794	414.4637	-2.9332	4.15E-10	4.69E-07	3403
Cluster-28897.15307	9.60353	87.66537	-3.211	3.68E-07	0.000157	531
Cluster-28897.16850	27.51831	442.0392	-4.0121	8.27E-06	0.001703	1778
Cluster-28897.16851	33.44889	396.6818	-3.5576	1.31E-07	6.71E-05	2297
Cluster-28897.17035	20.64831	217.2402	-3.3785	0.000565	0.039472	681
Cluster-28897.1713	20.37081	182.0789	-3.1593	1.20E-07	6.22E-05	2553
Cluster-28897.17455	62.51325	504.7856	-3.0026	5.39E-07	0.000214	1510
Cluster-28897.17670	42.17918	144.8436	-1.7879	1.49E-05	0.002664	885
Cluster-28897.17673	13.59242	165.2587	-3.5815	9.66E-08	5.31E-05	2327
Cluster-28897.17806	17.28856	70.70549	-2.0466	0.000276	0.023565	1651
Cluster-28897.17965	4.913639	107.0322	-4.4016	5.23E-05	0.007019	1082
Cluster-28897.18187	45.40324	462.9135	-3.3401	3.84E-06	0.000994	1959
Cluster-28897.18297	6.313828	122.1467	-4.2528	4.19E-05	0.005934	654
Cluster-28897.18866	49.51282	277.4366	-2.4804	1.94E-05	0.003253	1706
Cluster-28897.18898	11.27582	121.7751	-3.4	1.60E-05	0.002841	591
Cluster-28897.18900	11.32147	148.4228	-3.6941	1.86E-05	0.003165	499
Cluster-28897.18901	13.19972	198.4636	-3.881	0.000125	0.013222	910
Cluster-28897.18902	10.99801	84.21657	-2.9063	0.000401	0.030703	318
Cluster-28897.18903	1.473394	68.00322	-5.387	2.74E-06	0.000776	360
Cluster-28897.18904	11.48876	85.12108	-2.8779	7.28E-05	0.009098	449
Cluster-28897.18906	118.2364	536.2576	-2.185	8.80E-05	0.010272	710
Cluster-28897.18907	8.439556	196.0781	-4.5162	0.00055	0.03883	1070
Cluster-28897.18913	44.90573	334.8501	-2.8914	0.000226	0.020299	859
Cluster-28897.18914	5.553589	97.02494	-4.0897	0.000221	0.020023	726

Cluster-28897.18956	54.5034	345.553	-2.6558	0.000745	0.046864	774
Cluster-28897.18957	25.56375	132.2861	-2.3786	5.99E-05	0.007829	511
Cluster-28897.18961	15.73355	117.2841	-2.9036	8.26E-06	0.001703	359
Cluster-28897.18962	46.04103	169.4138	-1.8849	0.000299	0.024999	610
Cluster-28897.18967	1.389073	93.27309	-5.9337	4.38E-05	0.00613	753
Cluster-28897.18968	13.04085	137.4596	-3.3759	0.000657	0.043379	743
Cluster-28897.18970	20.66911	192.0405	-3.2011	0.000398	0.030539	605
Cluster-28897.19611	17.40021	314.2038	-4.1473	3.16E-07	0.00014	930
Cluster-28897.19613	69.64024	617.5324	-3.1469	0.000106	0.011789	620
Cluster-28897.19614	35.37564	365.7396	-3.3617	3.13E-06	0.000849	1037
Cluster-28897.19616	0.555629	190.7666	-8.216	4.03E-06	0.001031	762
Cluster-28897.19618	88.18838	591.9777	-2.7493	5.99E-09	4.99E-06	1478
Cluster-28897.19620	22.07223	210.5788	-3.2463	2.83E-10	3.29E-07	641
Cluster-28897.19622	6.25571	269.4824	-5.3983	0.0001	0.011359	1592
Cluster-28897.19623	3.223251	206.9809	-5.9541	2.16E-09	2.07E-06	901
Cluster-28897.20663	137.6333	508.6904	-1.8913	0.00059	0.040508	836
Cluster-28897.21298	1678.011	5500.446	-1.7128	3.45E-06	0.000909	2449
Cluster-28897.22934	8.124506	85.90072	-3.3803	2.85E-05	0.004346	1136
Cluster-28897.22994	79.62665	298.1998	-1.8995	3.22E-06	0.000866	2372
Cluster-28897.23018	5.917075	69.29374	-3.4916	0.000416	0.031479	1316
Cluster-28897.23019	73.65731	363.8293	-2.308	1.19E-07	6.22E-05	2581
Cluster-28897.23227	3.754028	46.77628	-3.613	2.96E-05	0.004452	600
Cluster-28897.23663	102.842	275.4729	-1.4185	0.000643	0.042856	917
Cluster-28897.23665	55.46322	191.2574	-1.781	0.000102	0.011523	964

Cluster-28897.24241	32.08332	387.9511	-3.5893	8.87E-06	0.00177	1121
Cluster-28897.24249	21.33827	159.7493	-2.8989	0.000118	0.012639	471
Cluster-28897.24259	22.383	223.791	-3.3051	7.34E-05	0.00912	520
Cluster-28897.24404	12.56954	107.7721	-3.0724	0.000372	0.029079	633
Cluster-28897.24410	15.61874	112.9522	-2.8388	4.48E-06	0.001111	719
Cluster-28897.24411	3.006256	55.41418	-4.1703	0.000301	0.025041	331
Cluster-28897.24413	19.38246	79.28754	-2.0182	0.000114	0.012362	442
Cluster-28897.24418	56.35028	215.8309	-1.927	0.000448	0.033333	1211
Cluster-28897.24869	37.40992	621.9173	-4.0472	0.00019	0.018059	1945
Cluster-28897.25215	20.12316	184.0935	-3.1756	5.53E-05	0.007359	2900
Cluster-28897.25218	16.05273	291.7813	-4.1742	7.04E-07	0.000269	676
Cluster-28897.25293	0	50.63103	-7.9863	9.67E-06	0.001904	976
Cluster-28897.25297	21.13073	177.5879	-3.0505	0.000166	0.016312	630
Cluster-28897.26103	3.716709	33.8275	-3.1654	0.000445	0.033162	383
Cluster-28897.26189	18.33877	135.2793	-2.8624	0.000287	0.02421	690
Cluster-28897.26330	250.0348	578.7729	-1.2105	0.000241	0.021297	2940
Cluster-28897.26475	3.137579	56.01757	-4.1528	9.85E-05	0.011218	1201
Cluster-28897.26525	16.36922	105.9538	-2.6783	0.000565	0.039472	2251
Cluster-28897.26643	515.5958	1571.395	-1.6074	0.000576	0.039988	1982
Cluster-28897.27048	0	15.33814	-6.2655	0.000701	0.045037	541
Cluster-28897.27199	15.11282	185.5762	-3.6034	8.88E-06	0.00177	1085
Cluster-28897.27200	17.16939	294.8108	-4.0802	4.32E-06	0.001093	1246
Cluster-28897.27203	3.19975	51.85121	-3.9488	1.27E-05	0.002341	340
Cluster-28897.27205	24.51625	173.4481	-2.8436	6.18E-05	0.008026	531

Cluster-28897.27206	13.75693	150.8294	-3.445	4.38E-06	0.001099	613
Cluster-28897.27275	5.277125	108.0654	-4.3104	0.000448	0.033333	1091
Cluster-28897.27549	124.7619	407.6768	-1.7036	9.93E-05	0.011266	2668
Cluster-28897.28354	19.75269	410.0654	-4.3594	3.18E-06	0.000857	2037
Cluster-28897.28993	256.2485	1153.269	-2.1715	1.06E-06	0.000374	3772
Cluster-28897.29028	11.70013	297.0011	-4.6379	7.68E-07	0.000288	1214
Cluster-28897.29712	16.95128	70.89247	-2.0743	0.000151	0.015234	699
Cluster-28897.29802	216.4195	529.476	-1.2889	0.000219	0.019909	1518
Cluster-28897.35257	74.39112	209.5782	-1.4942	1.98E-05	0.003313	2255
Cluster-28897.35643	0	24.69656	-6.9509	2.16E-05	0.003521	777
Cluster-28897.4727	1.557715	47.35527	-4.8088	1.80E-06	0.000574	3892
Cluster-28897.9233	16.20779	269.7615	-4.0903	7.96E-10	8.11E-07	3704
Cluster-31639.1	21.20146	126.5927	-2.5742	4.11E-07	0.000169	2447
Cluster-48158.1	13.87857	86.2602	-2.6256	0.000142	0.014555	2007
Cluster-48158.6	46.92734	226.9566	-2.2874	0.00011	0.012106	2071
Cluster-50448.0	10.5683	82.54635	-2.9831	2.37E-07	0.000112	1471
Cluster-52376.1	5.827351	101.163	-4.1375	3.52E-09	3.16E-06	2205
Cluster-56734.0	0.747772	59.39956	-6.3736	1.16E-05	0.002189	714
Cluster-56734.1	1.473394	158.2738	-6.6193	7.82E-08	4.55E-05	1323
Cluster-28897.25792	83.28141	547.6439	-2.7177	7.87E-09	6.43E-06	2123
Cluster-28897.24003	39.35659	248.2805	-2.6537	4.01E-07	0.000166	1113
Cluster-28897.14548	22.58513	99.08739	-2.1458	6.42E-06	0.001426	440
Cluster-28897.29030	32.27434	211.2719	-2.7149	3.35E-05	0.004899	1833
Cluster-28897.16999	174.9867	481.7664	-1.4583	0.0002	0.018722	2749

Cluster-28897.14551	337.8188	765.0629	-1.1806	0.000243	0.021395	2745
Cluster-48189.0	22.31272	105.178	-2.2308	0.000303	0.025136	2396
Cluster-28897.17977	32.22193	143.2427	-2.1619	0.000699	0.045007	2469
**ubiquitin**						
Cluster-41733.0	2.111993	74.55918	-5.1333	1.38E-09	1.37E-06	1925
Cluster-57141.0	0.724271	46.65143	-6.0217	6.58E-06	0.001447	1739
Cluster-28897.14794	146.5753	440.6943	-1.5913	9.92E-06	0.001933	1260
Cluster-28897.14793	74.57695	449.5012	-2.5841	0.000102	0.011495	1271
Cluster-28897.12985	179.9897	680.5843	-1.9197	0.000103	0.011579	3792
Cluster-28897.16999	174.9867	481.7664	-1.4583	0.0002	0.018722	2749
Cluster-28897.27073	126.8843	511.9925	-2.0072	0.000556	0.03908	3353
Cluster-28897.27074	70.20368	313.4276	-2.1493	0.000572	0.039839	3353
Cluster-28897.25170	0.362135	20.6893	-5.7364	0.000629	0.042328	789
**calcium**						
Cluster-28897.25792	83.28141	547.6439	-2.7177	7.87E-09	6.43E-06	2123
Cluster-28897.24003	39.35659	248.2805	-2.6537	4.01E-07	0.000166	1113
Cluster-28897.16599	28.25473	592.9032	-4.387	4.68E-07	0.000189	2351
Cluster-28897.16727	0.917765	64.83994	-6.0032	1.70E-06	0.000551	1276
Cluster-28897.16862	28.84768	151.3783	-2.3798	6.88E-06	0.00149	2222
Cluster-28897.25787	197.188	531.8668	-1.4308	1.63E-05	0.002855	4148
Cluster-28897.17425	3128.425	8644.069	-1.4665	0.00014	0.014387	2637
Cluster-28897.21375	0	19.6022	-6.6193	0.000153	0.015374	610
Cluster-28897.29137	0.469957	60.90441	-7.2921	0.000664	0.043652	1068
Cluster-28897.17977	32.22193	143.2427	-2.1619	0.000699	0.045007	2469

Cluster-28897.17528	233.5458	588.4664	-1.3347	0.000806	0.049815	782

Supplementary Table S5. Identification of resistance genes in up-regulated DEGs from Proot vs Croot

gene_id	PRoot_readcount	CRoot_readcount	log2FoldChange	pval	padj	Gene Length
**disease resistance protein**						
Cluster-28897.16022	142.7111	52.18832	1.4518	0.000345	0.012937	1191
Cluster-28897.2610	74.03323	13.885	2.4218	1.79E-05	0.001069	429
Cluster-28897.9627	100.1165	5.898579	4.071	1.85E-08	2.43E-06	446
Cluster-28897.13676	116.5276	4.862562	4.5835	5.82E-12	1.63E-09	1006
Cluster-28897.37476	13.24816	0.291069	5.342	0.000572	0.019589	394
Cluster-28897.3346	244.5547	59.14575	2.0511	5.46E-05	0.002801	3281
Cluster-28897.13683	170.4539	28.57041	2.5783	8.16E-05	0.003921	1558
Cluster-31868.0	9.465121	0	5.8179	0.001178	0.035041	585
Cluster-28897.32049	10.39687	0	5.9546	0.00103	0.031556	406
Cluster-28897.2493	328.0246	99.34349	1.7246	2.35E-07	2.36E-05	2960
Cluster-28897.14538	382.7888	131.3249	1.5433	0.001544	0.043486	1910
Cluster-28897.25057	574.2231	208.0752	1.4652	0.001634	0.045419	2717
Cluster-28897.13668	51.83705	9.225665	2.483	0.0008	0.025744	748
Cluster-28897.18718	85.86851	33.14128	1.3746	0.000921	0.028948	871
Cluster-28897.15330	120.8144	44.02398	1.4577	0.000928	0.029129	892
Cluster-28897.15332	54.15346	14.03609	1.9495	0.000474	0.016837	732
Cluster-28897.15322	54.79151	14.04635	1.9661	0.001784	0.048391	1105
Cluster-28897.16585	20.62434	0	6.9419	4.22E-06	0.000303	851

Cluster-28897.18717	87.7812	37.49852	1.2261	0.001384	0.039709	886
Cluster-50441.0	9.495859	0	5.8222	0.001067	0.032446	361
Cluster-32564.0	21.38782	0	6.994	2.96E-06	0.000221	1005
Cluster-28897.18143	30.69491	2.164276	3.8377	0.000173	0.007358	586
Cluster-28897.24023	94.05142	38.35222	1.2977	0.000866	0.027533	2109
Cluster-28897.19246	235.8579	56.3735	2.0661	2.05E-05	0.0012	1437
Cluster-28897.19248	62.79932	14.54357	2.104	0.001521	0.042935	580
Cluster-28897.2608	71.41306	26.38855	1.4406	0.001675	0.046333	1026
Cluster-55125.1	23.17187	0	7.1095	4.06E-06	0.000295	995
Cluster-28897.28650	72.27559	17.91369	2.0203	2.02E-05	0.001185	1642
Cluster-28897.2308	522.0653	8.557918	5.9353	4.87E-27	1.74E-23	1370
Cluster-28897.1961	122.6488	7.942695	3.9661	1.36E-07	1.47E-05	831
Cluster-28897.17943	31.49461	8.390326	1.9017	0.001711	0.047077	786
Cluster-28897.15	13.99571	0	6.3824	6.61E-05	0.00329	689
Cluster-28897.2740	73.14382	1.826546	5.3274	1.29E-12	4.19E-10	2166
Cluster-29110.0	45.07636	0.291069	7.1074	2.09E-07	2.13E-05	830
Cluster-28897.13092	30.79489	7.600183	2.0197	0.001342	0.038794	444
Cluster-49148.0	10.47316	0	5.9644	0.000493	0.017337	741
Cluster-28897.2636	768.751	4.192965	7.508	5.98E-33	1.07E-28	1687
Cluster-28897.3912	27.27575	4.1507	2.7007	0.000228	0.009209	866
Cluster-28897.2638	92.02032	9.523367	3.289	2.52E-06	0.000194	369
Cluster-28897.30227	142.4693	38.46251	1.8911	5.37E-07	4.93E-05	4110
Cluster-28897.30232	68.61552	16.24224	2.075	2.69E-05	0.001519	643
Cluster-28897.2303	18.46026	0.333333	5.8207	8.47E-05	0.004046	447

Cluster-28897.13672	307.2147	50.98649	2.5933	1.58E-05	0.000962	1195
Cluster-28897.13670	109.4725	12.90298	3.0911	0.000393	0.014465	554
Cluster-28897.14366	353.8843	125.5111	1.4973	0.001722	0.047254	2300
Cluster-28897.34484	95.65581	28.08938	1.766	1.98E-05	0.001166	3058
Cluster-31306.0	61.57018	0	8.5201	2.25E-10	4.61E-08	1839
Cluster-35591.0	32.22052	0.915471	5.1436	1.77E-06	0.000142	1249
Cluster-28897.14678	187.1486	43.99128	2.0935	7.37E-07	6.51E-05	1072
Cluster-28897.14706	37.74776	8.10697	2.233	0.00046	0.016401	628
Cluster-28897.11873	82.95346	7.394722	3.4751	9.47E-08	1.07E-05	1737
Cluster-28897.11877	23.43464	0	7.1261	8.18E-07	7.11E-05	769
Cluster-28897.3347	28.37259	3.898965	2.8559	0.000261	0.010327	1920
Cluster-28897.22554	65.67019	8.643913	2.9311	7.71E-05	0.003746	1225
Cluster-28897.22555	153.3207	51.93304	1.5605	0.000242	0.0097	1577
Cluster-28897.22556	61.50574	9.389246	2.7199	0.000955	0.029816	917
Cluster-28897.28263	38.39285	6.197361	2.6433	9.56E-05	0.004483	1122
Cluster-28897.21609	28.27603	3.366419	3.0741	0.000646	0.021596	1328
Cluster-28897.11728	133.7356	54.82128	1.2878	0.000442	0.015956	2048
Cluster-28897.32184	108.2325	41.55945	1.3811	0.000622	0.020979	2697
Cluster-28897.35561	350.5084	101.7934	1.7857	1.52E-08	2.03E-06	3242
Cluster-28897.15636	21.59025	0.914006	4.5666	0.000165	0.007082	703
Cluster-47579.0	74.56263	1.579207	5.6057	1.06E-11	2.88E-09	1441
Cluster-28897.1089	31.5208	4.033086	2.9824	2.75E-05	0.001549	573
**pathogenesis**						
Cluster-14190.0	41.76717	0.289603	6.9987	4.54E-06	0.000323	860

Cluster-10947.0	49.33415	0.873207	5.7827	7.75E-06	0.000516	806
Cluster-1038.0	38.17451	0	7.83	1.25E-05	0.000788	460
Cluster-24320.0	77.69539	0.95627	6.3726	0.000127	0.00567	1939
Cluster-18442.0	40.59929	0.333333	6.9569	0.000379	0.014013	1089
Cluster-28897.2378	45.03331	3.370816	3.7433	0.000599	0.020318	1067
Cluster-28897.10529	84.09013	21.97585	1.9408	0.00121	0.035829	969
Cluster-20754.0	37.3848	4.65016	3.0161	0.001259	0.036956	1001
Cluster-28897.19606	351.0307	100.5215	1.8062	0.001033	0.03163	2156
Cluster-30467.2	34.54947	3.283356	3.3841	1.78E-05	0.001065	2592
Cluster-28897.23252	427.9421	212.4022	1.0115	0.000387	0.014276	1878
Cluster-28897.21565	829.1966	237.143	1.8069	6.45E-05	0.003222	1076
Cluster-28897.7170	323.1784	4.060309	6.2785	1.49E-22	2.35E-19	2198
Cluster-28897.27769	117.2565	26.12617	2.1662	2.48E-06	0.000192	1138
**protein kinase**						
Cluster-35284.0	48.9457	0	8.1887	6.23E-10	1.14E-07	733
Cluster-28897.10337	1219.366	463.9211	1.3944	6.58E-10	1.20E-07	1752
Cluster-37298.0	49.92299	0	8.2173	1.81E-09	2.99E-07	1693
Cluster-28897.17251	175.7694	22.41863	2.9786	5.47E-09	8.13E-07	1530
Cluster-28897.21252	365.3038	33.50462	3.453	1.28E-07	1.39E-05	1366
Cluster-41400.2	76.19298	13.58954	2.4966	1.89E-07	1.95E-05	1573
Cluster-28897.15074	192.9028	54.52644	1.8257	2.17E-07	2.20E-05	2451
Cluster-28897.36736	53.81307	2.030155	4.6921	2.64E-07	2.62E-05	645
Cluster-28897.1711	49.78545	1.580672	5.0134	2.94E-07	2.88E-05	1403
Cluster-28897.1185	455.0212	169.2687	1.4274	3.71E-07	3.54E-05	2258

Cluster-28897.5431	165.829	41.50184	2.0017	7.69E-07	6.74E-05	1302
Cluster-28897.24967	32.18296	0.333333	6.622	1.20E-06	0.000101	474
Cluster-28897.13490	22.64155	0	7.0769	2.05E-06	0.000163	713
Cluster-28897.1714	66.34121	4.360557	3.9404	3.95E-06	0.000288	1071
Cluster-28897.1064	102.9313	0.624402	7.3883	7.40E-06	0.000498	1211
Cluster-28897.7985	216.7994	42.27872	2.3633	7.61E-06	0.000509	1164
Cluster-28897.13491	61.22566	10.60311	2.5428	7.84E-06	0.00052	1261
Cluster-28897.18072	192.2238	62.90836	1.614	9.99E-06	0.000642	4509
Cluster-28897.28411	35.96827	1.333333	4.8326	1.51E-05	0.000924	696
Cluster-45864.0	111.2119	1.493212	6.2112	1.95E-05	0.001152	2100
Cluster-48073.0	560.2903	269.3073	1.0575	2.41E-05	0.001382	2408
Cluster-28897.4548	27.9527	1.830942	3.9372	4.23E-05	0.002237	1673
Cluster-28897.3881	21.27853	0.622937	5.1239	8.70E-05	0.004143	596
Cluster-28897.33476	284.8368	117.6157	1.2764	0.000102	0.004712	900
Cluster-28897.26194	351.0205	101.4497	1.7923	0.000109	0.004996	2393
Cluster-28897.24869	44.92812	8.597252	2.3921	0.000145	0.006361	1945
Cluster-28897.28891	95.79675	32.75504	1.5453	0.000159	0.00688	1611
Cluster-28897.27667	207.8073	54.08837	1.9458	0.000177	0.007495	3739
Cluster-28897.25841	26.12106	2.16281	3.6055	0.000381	0.014071	861
Cluster-28897.401	37.79102	3.248804	3.5727	0.000445	0.016039	1616
Cluster-28897.30445	11.17061	0	6.0574	0.00047	0.016735	699
Cluster-52784.0	60.35403	12.64538	2.2681	0.000555	0.019106	2188
Cluster-28897.21609	28.27603	3.366419	3.0741	0.000646	0.021596	1328
Cluster-5940.0	15.10622	0	6.4923	0.000647	0.021609	932

Cluster-28897.30446	101.845	25.51133	2.0022	0.000954	0.029787	2057
Cluster-28897.35956	14.9286	0.580672	4.6395	0.000957	0.029857	1211
Cluster-45217.0	90.99861	2.897499	4.961	0.000989	0.030685	1836
Cluster-28897.19606	351.0307	100.5215	1.8062	0.001033	0.03163	2156
Cluster-28897.24064	335.9776	135.1258	1.3153	0.001053	0.032107	779
Cluster-44732.0	48.6515	0.582138	6.3578	0.001098	0.033132	1206
Cluster-28897.17455	135.9284	38.86672	1.8099	0.001168	0.034806	1510
Cluster-51933.0	54.33448	1.247339	5.4578	0.00117	0.034867	1201
Cluster-28897.25838	28.58754	5.03162	2.5255	0.001307	0.03803	592
Cluster-28897.5750	139.3393	66.57317	1.0683	0.00138	0.039611	2383
Cluster-28897.5442	66.49812	23.28119	1.5172	0.001773	0.048208	611
**ubiquitin**						
Cluster-28897.34662	241.6196	50.39124	2.263	7.55E-10	1.35E-07	722
Cluster-28897.11973	130.0224	11.92287	3.4496	2.22E-08	2.85E-06	874
Cluster-28897.22582	2477.985	930.6532	1.4129	2.15E-07	2.18E-05	509
Cluster-28897.21176	4215.8	1623.901	1.3763	1.40E-06	0.000116	1046
Cluster-28897.21955	3714.19	1581.857	1.2314	3.37E-05	0.00183	1884
Cluster-28897.20691	349.875	94.86331	1.8846	7.90E-05	0.003821	503
Cluster-28897.14487	162.6294	51.27548	1.6687	0.000119	0.005367	1963
Cluster-28897.928	47.99845	1.202143	5.3141	0.000169	0.007219	1031
Cluster-28897.204	53.59263	13.13088	2.0306	0.000472	0.016775	1684
Cluster-28897.13876	79.75743	20.61267	1.9487	0.001234	0.036395	615
Cluster-28897.21654	1927.592	931.5699	1.0494	0.001307	0.03803	1099
Cluster-37026.0	270.1831	110.3965	1.2934	0.001324	0.038405	520

Cluster-28897.34551	114.5993	7.042337	4.0191	0.001333	0.038585	441
Cluster-28897.12985	241.7791	95.98008	1.3349	0.001626	0.045233	3792
**calcium**						
Cluster-28897.17353	142.9746	5.527764	4.7031	2.75E-18	2.05E-15	1210
Cluster-28897.34662	241.6196	50.39124	2.263	7.55E-10	1.35E-07	722
Cluster-28897.11973	130.0224	11.92287	3.4496	2.22E-08	2.85E-06	874
Cluster-28897.22265	123.4239	34.4096	1.8392	1.72E-05	0.001035	2292
Cluster-28897.32188	654.711	121.4337	2.4305	6.30E-05	0.003164	3906
Cluster-28897.32152	6098.783	1718.532	1.8275	0.000108	0.004956	2684
Cluster-28897.15636	21.59025	0.914006	4.5666	0.000165	0.007082	703
Cluster-28897.36923	104.6585	13.06247	3.0021	0.000419	0.015254	401
Cluster-28897.977	21.84528	0.873207	4.6079	0.000452	0.016221	783
Cluster-55914.1	22.59351	0	7.0741	0.000501	0.017533	1005
Cluster-28897.29677	250.0592	83.73675	1.5764	0.000545	0.018813	1750
Cluster-28897.9966	1459.981	161.9829	3.1721	0.000785	0.025358	375
Cluster-28897.8313	86.80641	24.45172	1.8323	0.00091	0.028676	310
Cluster-28897.2083	14.78539	0	6.4613	0.00114	0.034121	760
Cluster-44987.0	22.42432	2.782816	3.0223	0.001225	0.036209	1256
Cluster-28897.13876	79.75743	20.61267	1.9487	0.001234	0.036395	615
Cluster-38553.0	18.85282	1.86881	3.3537	0.001458	0.041456	1003
Cluster-28897.33170	121.8834	39.37327	1.6261	0.001765	0.048045	2653

Supplementary Table S6. Identification of resistance genes in down-regulated DEGs from Proot vs Croot

gene_id	Proot_readcount	Croot_readcount	log2FoldChange	pval	padj	Gene Length
**disease resistance protein**						
Cluster-41093.0	0	37.41577	-7.5995	6.91E-07	6.14E-05	1028
Cluster-28897.9580	8.856469	85.5107	-3.2772	1.49E-06	0.000122	1927
Cluster-28897.31080	4.503265	54.827	-3.5997	3.71E-06	0.000273	1131
Cluster-28897.2787	15.40428	263.5229	-4.0968	8.69E-06	0.000569	4031
Cluster-42681.0	6.687841	83.07979	-3.6372	3.62E-05	0.001951	2052
Cluster-28897.4950	7.330641	56.94661	-2.9586	5.54E-05	0.002834	3465
Cluster-28897.17602	232.6644	580.4327	-1.3188	9.78E-05	0.00457	1803
Cluster-42888.0	3.788734	37.64252	-3.3038	0.000127	0.00567	2247
Cluster-28897.31738	2.087802	31.121	-3.894	0.000131	0.005836	1461
Cluster-28897.1962	14.61346	86.6813	-2.5651	0.000176	0.007449	1350
Cluster-28897.968	4.940222	34.4096	-2.8068	0.00029	0.011306	1424
Cluster-28897.17896	5.23487	36.16149	-2.7894	0.000344	0.01292	1216
Cluster-28897.9546	11.08522	60.50969	-2.4467	0.000436	0.01575	1420
Cluster-28897.27483	0.690621	17.12316	-4.6163	0.000493	0.017337	1099
Cluster-28897.16947	46.69304	165.1281	-1.8225	0.000653	0.021775	1840
Cluster-28897.5968	0	11.60311	-5.9057	0.000758	0.024665	718
Cluster-28897.5520	25.11053	75.15238	-1.5816	0.000836	0.026716	1752
Cluster-28897.24892	30.48202	80.02489	-1.3926	0.000844	0.02692	2736

Cluster-28897.17160	6.335128	34.97731	-2.4698	0.000847	0.026989	1328
Cluster-28897.11770	74.88005	170.3165	-1.1868	0.000923	0.028999	1652
Cluster-28897.31739	7.700429	37.33926	-2.2767	0.001017	0.031271	872
Cluster-41380.0	0	10.13381	-5.7154	0.001232	0.036344	633
**pathogenesis**						
Cluster-28897.5741	46.75428	584.0385	-3.6424	2.80E-17	1.86E-14	2265
Cluster-43683.0	5.584165	188.804	-5.0784	6.76E-15	3.09E-12	1500
Cluster-28897.18316	9.094192	115.1936	-3.6626	8.43E-14	3.33E-11	410
Cluster-28897.33994	40.01431	686.2367	-4.1009	1.67E-12	5.27E-10	4850
Cluster-28897.34508	0	143.8821	-9.5416	7.84E-11	1.76E-08	1122
Cluster-28897.6612	142.9609	622.2323	-2.1221	2.45E-09	3.95E-07	1571
Cluster-44716.0	38.38737	256.96	-2.7448	2.51E-09	4.03E-07	2245
Cluster-28897.4727	1.409703	60.54727	-5.4314	1.09E-07	1.20E-05	3892
Cluster-23268.0	3.809226	59.45957	-3.9579	2.17E-06	0.000172	465
Cluster-19099.0	0	23.69134	-6.9386	2.69E-06	0.000205	992
Cluster-28897.30018	854.5831	2670.553	-1.6439	3.56E-06	0.000262	2984
Cluster-28897.10204	19.16796	113.3679	-2.5614	4.74E-06	0.000335	1637
Cluster-28897.31169	0	23.71671	-6.942	8.42E-06	0.000555	414
Cluster-28897.5440	38.81159	139.9246	-1.8502	1.28E-05	0.000801	1104
Cluster-28897.5148	108.2029	412.4912	-1.9308	1.45E-05	0.000893	2019
Cluster-28897.34334	138.947	605.4146	-2.1239	1.55E-05	0.000944	3963
Cluster-28897.32673	63.87547	328.2185	-2.3608	2.59E-05	0.00147	3243
Cluster-53193.0	9.465121	65.37103	-2.7904	2.99E-05	0.001647	2008
Cluster-28897.30104	258.0031	566.3391	-1.1345	3.34E-05	0.001817	2172

Cluster-28897.29453	113.0691	382.7328	-1.7594	5.32E-05	0.002732	1254
Cluster-28897.27196	295.4648	1025.985	-1.7961	5.40E-05	0.002773	1322
Cluster-28897.5428	8.005322	49.50623	-2.6277	5.54E-05	0.002834	470
Cluster-44293.0	1.407427	25.79283	-4.201	5.81E-05	0.002954	1272
Cluster-28897.32726	316.7129	1165.201	-1.8792	6.20E-05	0.00312	1447
Cluster-28897.34408	41.35456	135.3762	-1.7127	7.25E-05	0.003554	6623
Cluster-28897.19421	1.399457	29.08799	-4.3801	0.000111	0.005049	860
Cluster-28897.18905	29.7709	110.3381	-1.8914	0.000115	0.005205	2799
Cluster-28897.13462	22.397	89.13856	-1.9946	0.000146	0.006372	1447
Cluster-41774.0	0	14.25875	-6.2062	0.000168	0.0072	970
Cluster-28897.13461	11.57797	59.92046	-2.3748	0.00019	0.007942	1085
Cluster-28897.7594	1.373271	30.31065	-4.4515	0.000234	0.009419	699
Cluster-57825.0	59.92867	203.0652	-1.7617	0.000357	0.01333	1657
Cluster-28897.9671	1.714596	28.20661	-4.0273	0.000424	0.015375	801
Cluster-35492.0	16.52387	128.3482	-2.9587	0.00045	0.016138	2470
Cluster-28897.3388	1.373271	25.36834	-4.1948	0.000485	0.017111	947
Cluster-28897.16739	26.35879	95.58388	-1.8608	0.000503	0.017585	2072
Cluster-57321.1	4.526033	33.97962	-2.9083	0.000692	0.022853	859
Cluster-28897.10616	190.7823	632.5971	-1.7289	0.000974	0.030319	2239
Cluster-40282.0	56.87952	130.2097	-1.1967	0.001343	0.038826	2773
**protein kinase**						
Cluster-28897.7433	152.0125	552.5198	-1.862	4.38E-05	0.002308	730
Cluster-28897.22401	43.37405	136.7845	-1.6584	0.000304	0.011751	531
Cluster-28897.5797	85.12638	1079.81	-3.6659	2.42E-08	3.09E-06	1216

Cluster-28897.10823	12.9797	52.41978	-2.015	0.000982	0.030494	738
Cluster-34429.0	12.87268	231.5056	-4.1653	3.86E-19	3.45E-16	1097
Cluster-28897.7608	6.662789	175.4293	-4.7207	4.66E-16	2.56E-13	594
Cluster-28897.8946	188.884	3544.413	-4.2302	1.79E-15	8.75E-13	752
Cluster-28897.7330	22.471	523.9232	-4.5444	3.23E-15	1.52E-12	823
Cluster-28897.5997	14.24595	242.7279	-4.088	1.00E-14	4.46E-12	456
Cluster-54996.0	2.79322	115.1325	-5.3631	8.59E-14	3.37E-11	471
Cluster-28897.10028	1.761274	101.0752	-5.8499	2.06E-11	5.28E-09	748
Cluster-28897.4906	29.03724	477.5201	-4.042	3.97E-11	9.45E-09	732
Cluster-43446.0	1.419949	97.51586	-6.118	5.52E-11	1.28E-08	601
Cluster-28897.7607	22.86925	326.2967	-3.8364	7.28E-11	1.64E-08	658
Cluster-28897.32781	1.409703	85.17405	-5.9258	9.52E-11	2.12E-08	378
Cluster-28897.9242	39.53544	476.6227	-3.5932	2.37E-10	4.83E-08	593
Cluster-28897.6604	40.47197	798.4038	-4.3038	2.65E-10	5.27E-08	660
Cluster-28897.25754	224.4553	997.4386	-2.1517	4.17E-10	7.96E-08	776
Cluster-28897.7344	5.695737	135.5813	-4.5827	4.98E-10	9.35E-08	640
Cluster-28897.7786	1.401733	73.29976	-5.7114	8.12E-09	1.16E-06	830
Cluster-28897.8867	3.19944	88.45486	-4.8009	9.31E-09	1.31E-06	378
Cluster-28897.10276	554.1747	4162.342	-2.9091	4.20E-08	5.14E-06	733
Cluster-23908.0	6.638879	103.3615	-3.9607	1.08E-07	1.20E-05	391
Cluster-28897.8573	27.78898	200.7202	-2.8545	1.08E-07	1.20E-05	340
Cluster-28897.32706	0.708837	57.10703	-6.3477	2.79E-07	2.75E-05	1502
Cluster-28897.2758	3.152762	50.22172	-3.9931	3.04E-07	2.97E-05	728
Cluster-22342.0	3.457655	60.99721	-4.1332	3.49E-07	3.36E-05	537

Cluster-42194.0	2.449619	47.2049	-4.2705	3.99E-07	3.76E-05	588
Cluster-28897.10098	1.365301	49.59701	-5.16	6.91E-07	6.14E-05	1888
Cluster-16023.0	0	27.18455	-7.1364	7.47E-07	6.57E-05	413
Cluster-48375.0	22.38448	123.01	-2.4594	8.25E-07	7.16E-05	2122
Cluster-32916.0	3.794428	60.02698	-3.9733	1.03E-06	8.74E-05	315
Cluster-28897.8574	3.235872	106.2219	-5.0502	2.02E-06	0.000161	405
Cluster-28897.36461	0.719083	37.39263	-5.7327	2.49E-06	0.000192	448
Cluster-15914.0	0.349295	99.15849	-8.0414	6.86E-06	0.000467	1446
Cluster-24045.1	0.700867	34.33487	-5.6161	6.90E-06	0.000469	449
Cluster-53215.0	1.381241	38.8307	-4.8029	7.53E-06	0.000505	448
Cluster-18577.0	1.409703	32.94947	-4.5561	8.22E-06	0.000542	464
Cluster-48213.0	0	19.4973	-6.6556	1.36E-05	0.000846	600
Cluster-28897.7393	11.56658	58.88854	-2.3496	1.45E-05	0.000896	691
Cluster-28897.33638	8.533359	107.4596	-3.66	2.66E-05	0.001505	789
Cluster-28897.7829	4.883298	33.73514	-2.7857	2.74E-05	0.001545	382
Cluster-36193.0	2.775004	38.19558	-3.7775	2.79E-05	0.001563	382
Cluster-22342.1	0.359541	24.43853	-6.0196	2.92E-05	0.001624	709
Cluster-34425.0	2.407493	40.56531	-4.0634	6.11E-05	0.003083	670
Cluster-38395.0	0	19.6608	-6.6709	6.54E-05	0.003257	376
Cluster-27377.0	1.751029	29.67993	-4.0851	8.96E-05	0.004248	362
Cluster-19526.0	0	14.71595	-6.2506	9.71E-05	0.004543	522
Cluster-16178.0	3.11633	31.50177	-3.3284	0.000146	0.006365	420
Cluster-17229.0	0.359541	19.0853	-5.6634	0.000162	0.006992	452
Cluster-28897.1962	14.61346	86.6813	-2.5651	0.000176	0.007449	1350

Cluster-28897.10657	0.682651	21.11252	-4.9231	0.000235	0.009451	334
Cluster-28897.7847	5.724199	61.98918	-3.4448	0.000292	0.011338	416
Cluster-48034.0	20.65849	84.81457	-2.0387	0.000339	0.012811	2392
Cluster-46598.0	4.521481	28.08537	-2.6298	0.00037	0.013762	453
Cluster-28897.17016	43.54234	165.5341	-1.925	0.0004	0.014656	2479
Cluster-28897.33222	0	11.38593	-5.88	0.000489	0.017225	514
Cluster-28897.33528	30.95655	90.5791	-1.5508	0.000511	0.01778	1759
Cluster-20664.0	0.359541	14.92873	-5.3078	0.00069	0.022816	356
Cluster-30539.0	3.129994	24.35693	-2.9536	0.000724	0.023735	321
Cluster-28897.35176	23.61907	125.0421	-2.4051	0.001115	0.033499	1478
**ubiquitin**						
Cluster-28897.8863	7.728891	145.2541	-4.236	3.56E-16	1.99E-13	1954
Cluster-28897.26945	677.5145	1905.161	-1.4914	1.00E-06	8.53E-05	2118
Cluster-18013.0	1.740783	33.04025	-4.2428	3.43E-06	0.000254	543
Cluster-28897.4605	26.71811	153.4167	-2.5205	8.37E-06	0.000551	1074
Cluster-18013.2	19.57759	99.06312	-2.3405	7.93E-05	0.003833	987
Cluster-28897.4607	7.661721	46.86047	-2.612	0.000199	0.008225	616
Cluster-18365.0	4.508959	47.4834	-3.3935	0.00037	0.013744	345
Cluster-28897.16507	27.69449	93.72324	-1.761	0.000584	0.019945	2080
Cluster-38917.0	1.050162	16.11838	-3.9429	0.001071	0.032523	327
Cluster-28897.10511	405.2019	851.3979	-1.0715	0.00132	0.038339	5319
Cluster-25787.1	21.45044	296.2739	-3.7904	6.25E-19	5.33E-16	1945
Cluster-28897.14337	11.16605	169.3158	-3.9213	6.82E-16	3.59E-13	401
Cluster-28897.34661	5.988108	91.25125	-3.9351	3.71E-11	8.91E-09	1090

Cluster-28897.13549	3.510027	68.99142	-4.3009	2.43E-10	4.92E-08	912
Cluster-28897.13863	81.95203	743.4999	-3.1809	2.53E-10	5.09E-08	1717
Cluster-28897.7994	16.59538	397.1361	-4.5785	8.15E-10	1.43E-07	1605
Cluster-28897.23882	192.6899	1085.369	-2.4937	4.61E-09	7.00E-07	2613
Cluster-28897.14348	31.80406	187.6987	-2.5613	1.52E-08	2.03E-06	409
Cluster-28897.10134	6.715161	104.23	-3.9624	1.55E-08	2.07E-06	1788
Cluster-28897.7219	0.349295	56.39263	-7.2285	7.53E-08	8.73E-06	512
Cluster-28897.22830	10.89645	251.9354	-4.5327	1.45E-07	1.55E-05	3157
Cluster-28897.29881	23.62818	336.4219	-3.8329	1.63E-07	1.71E-05	699
Cluster-28897.18234	19.77797	109.7748	-2.4758	1.47E-06	0.00012	952
Cluster-28897.27840	29.03133	97.58141	-1.7485	2.32E-06	0.000181	1011
Cluster-28897.14343	116.5826	349.2365	-1.5824	9.23E-06	0.0006	1007
Cluster-28897.14349	23.63821	110.7187	-2.2263	5.13E-05	0.00265	345
Cluster-28897.11974	27.04804	106.4365	-1.9741	0.00029	0.011302	771
Cluster-28897.26511	792.7493	2428.816	-1.6154	0.000325	0.012388	3171
Cluster-28897.14336	92.31044	338.4756	-1.8742	0.000636	0.021339	2722
Cluster-22747.0	0	13.48665	-6.1284	0.000696	0.022979	849
Cluster-28897.10684	9.463988	78.6503	-3.055	0.000827	0.026483	1483
Cluster-28897.14346	29.43641	86.06854	-1.5488	0.001358	0.039162	437
Cluster-28897.11972	50.70243	120.6332	-1.2496	0.00148	0.041983	810
Cluster-28897.23036	31.94409	92.15152	-1.531	0.001502	0.042511	356
Cluster-28897.14345	7.991658	38.47639	-2.2654	0.00165	0.045765	1267
Cluster-53060.0	5.297488	26.20445	-2.3132	0.001828	0.049236	3703
Cluster-20697.0	1.401733	43.69511	-4.9651	3.97E-07	3.75E-05	558

Cluster-43120.0	0	22.93428	-6.8924	2.63E-06	0.000202	1730
Cluster-28897.16485	7.387565	52.43134	-2.8324	9.72E-06	0.000626	802
Cluster-28897.16558	8.825731	54.14089	-2.6221	6.10E-05	0.003079	1554
Cluster-27667.0	0	10.9258	-5.822	0.000533	0.018462	473
Cluster-28897.23600	8.764255	38.80972	-2.1486	0.001766	0.048045	1774
Cluster-42842.0	1.751029	57.57834	-5.0419	1.35E-07	1.46E-05	1540
Cluster-28897.12162	32.37399	177.7339	-2.4564	8.33E-07	7.23E-05	1169
Cluster-28897.9867	1.761274	23.02799	-3.7129	0.00032	0.012227	739
Cluster-28897.25033	199.0471	662.5877	-1.7353	0.000392	0.014425	2787
Cluster-28897.5652	17.79583	63.90835	-1.8456	0.001368	0.039369	2493
Cluster-40116.0	0	19.66597	-6.6688	1.44E-05	0.00089	1136
Cluster-45339.0	28.80954	149.1807	-2.3729	0.000328	0.012471	2378
Cluster-28897.29123	242.2276	674.2633	-1.4772	0.000357	0.01333	1436
Cluster-33001.0	0.682651	21.91338	-4.9817	0.000397	0.014573	1420
Cluster-21660.0	0	19.85523	-6.6812	0.000623	0.02098	1287
Cluster-47752.0	13.63389	58.2339	-2.0963	0.000846	0.026989	1181
Cluster-28897.35176	23.61907	125.0421	-2.4051	0.001115	0.033499	1478
Cluster-42624.0	1.078624	23.87659	-4.4908	0.001254	0.036857	743
**calcium**						
Cluster-28897.8731	127.6787	2889.596	-4.5007	2.95E-21	3.77E-18	691
Cluster-25787.1	21.45044	296.2739	-3.7904	6.25E-19	5.33E-16	1945
Cluster-28897.14337	11.16605	169.3158	-3.9213	6.82E-16	3.59E-13	401
Cluster-28897.34661	5.988108	91.25125	-3.9351	3.71E-11	8.91E-09	1090
Cluster-28897.13549	3.510027	68.99142	-4.3009	2.43E-10	4.92E-08	912

Cluster-28897.13863	81.95203	743.4999	-3.1809	2.53E-10	5.09E-08	1717
Cluster-28897.7994	16.59538	397.1361	-4.5785	8.15E-10	1.43E-07	1605
Cluster-28897.23882	192.6899	1085.369	-2.4937	4.61E-09	7.00E-07	2613
Cluster-28897.14348	31.80406	187.6987	-2.5613	1.52E-08	2.03E-06	409
Cluster-28897.10134	6.715161	104.23	-3.9624	1.55E-08	2.07E-06	1788
Cluster-28897.7219	0.349295	56.39263	-7.2285	7.53E-08	8.73E-06	512
Cluster-28897.22830	10.89645	251.9354	-4.5327	1.45E-07	1.55E-05	3157
Cluster-28897.29881	23.62818	336.4219	-3.8329	1.63E-07	1.71E-05	699
Cluster-28897.18234	19.77797	109.7748	-2.4758	1.47E-06	0.00012	952
Cluster-28897.27840	29.03133	97.58141	-1.7485	2.32E-06	0.000181	1011
Cluster-28897.14343	116.5826	349.2365	-1.5824	9.23E-06	0.0006	1007
Cluster-28897.14349	23.63821	110.7187	-2.2263	5.13E-05	0.00265	345
Cluster-28897.11974	27.04804	106.4365	-1.9741	0.00029	0.011302	771
Cluster-28897.14336	92.31044	338.4756	-1.8742	0.000636	0.021339	2722
Cluster-28897.10684	9.463988	78.6503	-3.055	0.000827	0.026483	1483
Cluster-28897.14346	29.43641	86.06854	-1.5488	0.001358	0.039162	437
Cluster-28897.11972	50.70243	120.6332	-1.2496	0.00148	0.041983	810
Cluster-28897.14345	7.991658	38.47639	-2.2654	0.00165	0.045765	1267
Cluster-43683.0	5.584165	188.804	-5.0784	6.76E-15	3.09E-12	1500
Cluster-48478.0	23.45514	117.4051	-2.3243	9.32E-06	0.000604	2068
Cluster-28897.34408	41.35456	135.3762	-1.7127	7.25E-05	0.003554	6623
Cluster-28897.6437	6.331709	199.5224	-4.9821	3.26E-20	3.43E-17	616
Cluster-17492.1	0	34.32499	-7.4725	6.37E-08	7.50E-06	758
Cluster-28897.16742	12.57918	75.50715	-2.5843	2.34E-07	2.36E-05	5523

Cluster-28897.5450	0.700867	26.03979	-5.2189	3.86E-05	0.002058	354
Cluster-28897.19642	595.2336	1497.115	-1.3308	0.000106	0.004883	3178
Cluster-28897.11705	353.1085	835.9899	-1.2435	0.000264	0.010445	2018
Cluster-28897.5855	35.01715	291.5321	-3.0576	1.01E-09	1.76E-07	1570
Cluster-28897.8867	3.19944	88.45486	-4.8009	9.31E-09	1.31E-06	378
Cluster-28897.7762	73.34304	561.4172	-2.9368	2.38E-08	3.05E-06	1372
Cluster-23268.0	3.809226	59.45957	-3.9579	2.17E-06	0.000172	465
Cluster-28897.30527	44.46952	201.4891	-2.1779	4.36E-06	0.000311	1763
Cluster-28897.31750	460.8465	1164.048	-1.3369	2.97E-05	0.001643	1323
Cluster-28897.23013	76.99996	368.1309	-2.2579	4.69E-05	0.002443	803
Cluster-28897.30725	79.23667	255.7516	-1.6916	4.96E-05	0.002571	2102
Cluster-28897.8868	96.59802	534.1714	-2.4677	0.000133	0.005912	1037
Cluster-28897.20123	204.6515	530.7235	-1.375	0.000187	0.007834	3760
Cluster-50537.1	0	11.16982	-5.8565	0.001694	0.046678	644

Supplementary Table S7. DEGs enriched in the plant–pathogen interaction pathway in the leaves and roots from white clover after dodder parasitism

gene_id	KO ID	KO Name	KO Description	log2FC
P_{leaf} vs C_{leaf}				
Cluster-28897.22619	K13449	PR1	pathogenesis-related protein 1	-3.52
Cluster-28897.17987	K05391	CNGF	cyclic nucleotide gated channel, other eukaryote	-1.25
Cluster-28897.24820	K05391	CNGF	cyclic nucleotide gated channel, other eukaryote	-1.33
Cluster-28897.2637	K13457	RPM1, RPS3	disease resistance protein RPM1	-5.68
Cluster-28897.29775	K13457	RPM1, RPS3	disease resistance protein RPM1	-1.88
Cluster-28897.17528	K13448	CML	calcium-binding protein CML	-1.33
Cluster-28897.18898	K13420	FLS2	LRR receptor-like serine/threonine-protein kinase FLS2	-3.4
Cluster-28897.18907	K13420	FLS2	LRR receptor-like serine/threonine-protein kinase FLS2	-4.52
Cluster-28897.18900	K13420	FLS2	LRR receptor-like serine/threonine-protein kinase FLS2	-3.70
Cluster-28897.18901	K13420	FLS2	LRR receptor-like serine/threonine-protein kinase FLS2	-3.88
Cluster-8612.0	K02183	CALM	calmodulin	7.92
Cluster-37557.3	K04079	htpG,HSP90A	molecular chaperone HtpG	6.04
Cluster-28897.27133	K13448	CML	calcium-binding protein CML	1.85
Cluster-40098.0	K05391	CNGF	cyclic nucleotide gated channel, other eukaryote	5.53
P_{root} vs C_{root}				
Cluster-28897.3347	K13457	RPM1, RPS3	disease resistance protein RPM1	2.86
Cluster-28897.2608	K13457	RPM1, RPS3	disease resistance protein RPM1	1.44
Cluster-49148.0	K13457	RPM1, RPS3	disease resistance protein RPM1	5.96

Cluster-28897.2638	K13457	RPM1, RPS3	disease resistance protein RPM1	3.29
Cluster-28897.14538	K13457	RPM1, RPS3	disease resistance protein RPM1	1.54
Cluster-28897.2610	K13457	RPM1, RPS3	disease resistance protein RPM1	2.42
Cluster-28897.2308	K13457	RPM1, RPS3	disease resistance protein RPM1	5.94
Cluster-55125.1	K13457	RPM1, RPS3	disease resistance protein RPM1	7.11
Cluster-28897.3912	K13457	RPM1, RPS3	disease resistance protein RPM1	2.70
Cluster-28897.3346	K13457	RPM1, RPS3	disease resistance protein RPM1	2.05
Cluster-28897.1961	K13457	RPM1, RPS3	disease resistance protein RPM1	3.97
Cluster-28897.34484	K13457	RPM1, RPS3	disease resistance protein RPM1	1.77
Cluster-28897.2636	K13457	RPM1, RPS3	disease resistance protein RPM1	7.51
Cluster-28897.1962	K13457	RPM1, RPS3	disease resistance protein RPM1	-2.57
Cluster-41380.0	K13457	RPM1, RPS3	disease resistance protein RPM1	-5.72
Cluster-42888.0	K13457	RPM1, RPS3	disease resistance protein RPM1	-3.30
Cluster-28897.25841	K13414	MEKK1P	mitogen-activated protein kinase kinase kinase 1, plant	3.61
Cluster-28897.25838	K13414	MEKK1P	mitogen-activated protein kinase kinase kinase 1, plant	2.53
Cluster-35284.0	K13430	PBS1	serine/threonine-protein kinase PBS1	8.19
Cluster-28897.19336	K05391	CNGF	cyclic nucleotide gated channel, other eukaryote	-2.34
Cluster-28897.19337	K05391	CNGF	cyclic nucleotide gated channel, other eukaryote	-2.22
Cluster-28897.33433	K13447	RBOH	respiratory burst oxidase	-2.25
Cluster-28897.8868	K13447	RBOH	respiratory burst oxidase	-2.47
Cluster-28897.5855	K02183	CALM	calmodulin	-3.06

Supplementary Table S8. DEGs enriched in the plant hormone signal transduction pathway in white clover’s leaves and roots after dodder parasitism

	Gene ID	KO ID	KO	KO annotation	Log2FC
P_{leaf} vs C_{leaf}					
Auxin	Cluster-40615.0	K14488	SAUR	SAUR family protein	-6.60
Auxin	Cluster-28897.5110	K14487	GH3	auxin responsive GH3 gene family	3.30
CTK	Cluster-28897.19584	K14490	AHP	histidine-containing phosphotransfer peotein	-3.48
CTK	Cluster-32110.0	K14491	ARR-B	two-component response regulator ARR-B family	7.39
ABA	Cluster-28897.34756	K14498	SNRK2	serine/threonine-protein kinase SRK2	2.05
SA	Cluster-28897.22619	K13449	PR1	pathogenesis-related protein 1	-3.52
P_{root} vs C_{root}					
CTK	Cluster-32110.0	K14491	ARR-B	two-component response regulator ARR-B family	4.21
CTK	Cluster-28897.13647	K14491	ARR-B	two-component response regulator ARR-B family	3.59
CTK	Cluster-48564.0	K14492	ARR-A	two-component response regulator ARR-A family	-1.63
ET	Cluster-30903.0	K14516	ERF1	ethylene-responsive transcription factor 1	1.98
ET	Cluster-44732.0	K14512	MPK6	mitogen-activated protein kinase 6	6.36
Auxin	Cluster-28897.37861	K14486	ARF	auxin response factor	1.33
Auxin	Cluster-28897.6014	K14488	SAUR	SAUR family protein	-2.90
Auxin	Cluster-45618.0	K13946	AUX1, [illegible]	auxin influx carrier (AUX1 LAX family)	-1.51
ABA	Cluster-28897.5726	K14432	ABF	ABA responsive element binding factor	-3.94
ABA	Cluster-57825.0	K14498	SNRK2	serine/threonine-protein kinase SRK2	-1.76
SA	Cluster-28897.6383	K14431	TGA	transcription factor TGA	-2.71

SA	Cluster-28897.33887	K14431	TGA	transcription factor TGA	-2.16
JA	Cluster-28897.13549	K14506	JAR1	jasmonic acid-amino synthetase	-4.30

Supplementary Table S9. DEGs enriched in the phenylpropanoid biosynthesis pathway in the white clover leaves and roots after dodder parasitism

**Gene ID**	**KO ID**	**KO name**	**KO annotation**	**Log2FC**
P_{leaf} vs C_{leaf}				
Cluster-52394.0	K01188	E3.2.1.21 ,bglX, bglB	beta-glucosidase	-3.86
Cluster-28897.12824	K12355	E1.2.1.68, REF1	coniferyl-aldehyde dehydrogenase	-3.15
Cluster-28897.20416	K00430	E1.11.1.7, PRDX6	peroxidase	-2.21
Cluster-28897.25631	K01188	E3.2.1.21, bglX, bglB	beta-glucosidase	3.28
Cluster-28897.18603	K00588	E2.1.1.104, CCoAOMT	caffeoyl-CoA O-methyltransferase	-3.99
Cluster-28897.12905	K00083	E1.1.1.195, CAD	cinnamyl-alcohol dehydrogenase	1.48
Cluster-28897.21230	K00083	E1.1.1.195, CAD	cinnamyl-alcohol dehydrogenase	2.53
Cluster-28897.25606	K01188	E3.2.1.21 ,bglX, bglB	beta-glucosidase	3.43
Cluster-28897.25627	K01188	E3.2.1.21 ,bglX, bglB	beta-glucosidase	2.26
Cluster-33784.0	K11188	PRDX6	peroxiredoxin 6, 1-Cys peroxiredoxin	7.21
Cluster-28897.3521	K05349	E3.2.1.21, bglX	beta-glucosidase	4.05
Cluster-28897.25638	K01188	E3.2.1.21 ,bglX, bglB	beta-glucosidase	2.98
Cluster-28897.5861	K13066	E2.1.1.68, COMT	caffeic acid 3-O-methyltransferase	4.02
Cluster-28897.11757	K10775	E4.3.1.24, PAL	phenylalanine ammonia-lyase	1.84
Cluster-28897.25617	K01188	E3.2.1.21 ,bglX, bglB	beta-glucosidase	1.99
Cluster-28897.25635	K01188	E3.2.1.21 ,bglX, bglB	beta-glucosidase	2.15
P_{root} vs C_{root}				
Cluster-28897.37941	K01188	E3.2.1.21 ,bglX, bglB	beta-glucosidase	4.46
Cluster-28897.16469	K00083	E1.1.1.195, CAD	cinnamyl-alcohol dehydrogenase	-1.98
Cluster-14257.0	K01188	E3.2.1.21 ,bglX, bglB	beta-glucosidase	6.23
Cluster-57653.0	K13066	E2.1.1.68, COMT	caffeic acid 3-O-methyltransferase	-1.53
Cluster-28897.5019	K00430	E1.11.1.7, PRDX6	peroxidase	-2.29
Cluster-28897.21230	K00083	E1.1.1.195, CAD	cinnamyl-alcohol dehydrogenase	-1.11

Cluster-51220.0	K00083	E1.1.1.195, CAD	cinnamyl-alcohol dehydrogenase	-1.59
Cluster-45615.0	K05350	E3.2.1.21,bglB	beta-glucosidase	-2.61
Cluster-28897.21461	K00430	E1.11.1.7, PRDX6	peroxidase	-1.43
Cluster-28897.10004	K12356	E2.4.1.111, UGT72E	coniferyl-alcohol glucosyltransferase	-2.43
Cluster-28897.34522	K00430	E1.11.1.7, PRDX6	peroxidase	-1.77
Cluster-51096.0	K13066	E2.1.1.68, COMT	caffeic acid 3-O-methyltransferase	-6.16
Cluster-28897.3248	K12356	E2.4.1.111, UGT72E	coniferyl-alcohol glucosyltransferase	-1.77
Cluster-28897.22464	K00430	E1.11.1.7, PRDX6	peroxidase	-4.06
Cluster-28897.21232	K00083	E1.1.1.195, CAD	cinnamyl-alcohol dehydrogenase	-1.62
Cluster-28897.21343	K01188	E3.2.1.21,bglX, bglB	beta-glucosidase	-2.17
Cluster-28897.10573	K00430	E1.11.1.7, PRDX6	peroxidase	-2.76
Cluster-19366.0	K12356	E2.4.1.111, UGT72E	coniferyl-alcohol glucosyltransferase	-4.14
Cluster-28897.5566	K00430	E1.11.1.7, PRDX6	peroxidase	-1.58

Supplementary Table S10. The correlation coefficients of transcription factors and structural genes

Transcription factors	DEGs related to phenylpropanoid biosynthesis	Correlation coefficients
Cluster-28897.1112 (AP2/ERF-1)	Cluster-28897.10573,PRDX6-7	0.999124098
	Cluster-28897.10004,UGT72E-1	0.997394924
	Cluster-51096.0,COMT-3	0.994754773
	Cluster-28897.3248,UGT72E-2	0.988760816
	Cluster-28897.5019,PRDX6-3	0.985735375
	Cluster-28897.34522,PRDX6-5	0.984644183
	Cluster-45615.0,bglB	0.983001027
	Cluster-28897.5566,PRDX6-8	0.978966567
	Cluster-57653.0,COMT-2	0.977736061
	Cluster-51220.0,CAD-5	0.970814999
	Cluster-28897.21232,CAD-6	0.955061615
	Cluster-19366.0,UGT72E-3	0.914820935
Cluster-42284.0 (AP2/ERF-2)	Cluster-28897.10004,UGT72E-1	0.999586859
	Cluster-28897.10573,PRDX6-7	0.998947927
	Cluster-51096.0,COMT-3	0.994422906
	Cluster-28897.5019,PRDX6-3	0.992028054
	Cluster-45615.0,bglB	0.989608004
	Cluster-28897.34522,PRDX6-5	0.985239065
	Cluster-28897.3248,UGT72E-2	0.985092765
	Cluster-57653.0,COMT-2	0.982686542
	Cluster-28897.5566,PRDX6-8	0.979815246
	Cluster-51220.0,CAD-5	0.965388159
	Cluster-28897.21232,CAD-6	0.941366308
Cluster-28897.1396 (AP2/ERF-3)	Cluster-28897.12824,REF1	0.950965519
	Cluster-28897.18603,CCoAOMT	0.93484299
	Cluster-52394.0,bglX,bglB-7	0.900421841
	Cluster-28897.21230,CAD-2	-0.941856291
	Cluster-28897.21230,CAD-4	-0.941856291
Cluster-28897.15707 (AP2/ERF-4)	Cluster-52394.0,bglX,bglB-7	0.998634156
	Cluster-28897.12824,REF1	0.965022354
	Cluster-28897.18603,CCoAOMT	0.925022179

	Cluster-28897.21461,PRDX6-4	0.920586723
Cluster-28897.7026 (AP2/ERF-5)	Cluster-28897.37941,bglX,bglB-8	0.922313801
	Cluster-14257.0,bglX,bglB-9	0.917587583
	Cluster-28897.16469,CAD-3	-0.91925625
Cluster-28897.7769 (AP2/ERF-6)	Cluster-28897.37941,bglX,bglB-8	0.922313801
	Cluster-14257.0,bglX,bglB-9	0.917587583
	Cluster-28897.16469,CAD-3	-0.91925625
Cluster-30903.0 (AP2/ERF-7)	Cluster-28897.37941,bglX,bglB-8	0.968331908
	Cluster-14257.0,bglX,bglB-9	0.965842574
Cluster-28897.29534 （bHLH-1）	Cluster-52394.0,bglX,bglB-7	0.997321435
	Cluster-28897.12824,REF1	0.978700934
	Cluster-28897.18603,CCoAOMT	0.949379972
	Cluster-28897.21461,PRDX6-4	0.918657084
Cluster-28897.36821（bHLH-2）	Cluster-14257.0,bglX,bglB-9	0.934132771
	Cluster-28897.37941,bglX,bglB-8	0.933392543
Cluster-28897.9204（bHLH-3）	Cluster-51096.0,COMT-3	0.999243258
	Cluster-28897.3248,UGT72E-2	0.995348627
	Cluster-28897.10004,UGT72E-1	0.991033609
	Cluster-45615.0,bglB	0.987933202
	Cluster-51220.0,CAD-5	0.98702757
	Cluster-28897.5019,PRDX6-3	0.986194079
	Cluster-28897.10573,PRDX6-7	0.985012896
	Cluster-28897.34522,PRDX6-5	0.953385198
	Cluster-57653.0,COMT-2	0.951280413
	Cluster-28897.5566,PRDX6-8	0.944076681
	Cluster-28897.21232,CAD-6	0.942908986
	Cluster-19366.0,UGT72E-3	0.91493615
Cluster-44145.0（bHLH-4）	Cluster-28897.12824,REF1	0.976447809
	Cluster-28897.18603,CCoAOMT	0.974892531
	Cluster-52394.0,bglX,bglB-7	0.906722296
	Cluster-28897.21230,CAD-2	-0.916644584
	Cluster-28897.21230,CAD-4	-0.916644584
Cluster-47647.0 （bHLH-5）	Cluster-28897.37941,bglX,bglB-8	0.923174741

	Cluster-14257.0,bglX,bglB-9	0.918536466
	Cluster-28897.16469,CAD-3	-0.918332242
Cluster-24320.0（bZIP-1）	Cluster-14257.0,bglX,bglB-9	0.999867552
	Cluster-28897.37941,bglX,bglB-8	0.999513542
Cluster-28897.5726（bZIP-2）	Cluster-51096.0,COMT-3	0.99428908
	Cluster-45615.0,bglB	0.992529244
	Cluster-28897.5019,PRDX6-3	0.989411792
	Cluster-28897.10004,UGT72E-1	0.987206584
	Cluster-28897.3248,UGT72E-2	0.986351343
	Cluster-51220.0,CAD-5	0.977765652
	Cluster-28897.10573,PRDX6-7	0.976345951
	Cluster-57653.0,COMT-2	0.947082183
	Cluster-28897.34522,PRDX6-5	0.942246073
	Cluster-28897.5566,PRDX6-8	0.93255094
	Cluster-28897.21232,CAD-6	0.916593565
Cluster-49382.0（bZIP-3）	Cluster-51096.0,COMT-3	0.995763055
	Cluster-45615.0,bglB	0.995127577
	Cluster-28897.5019,PRDX6-3	0.992998691
	Cluster-28897.10004,UGT72E-1	0.991663521
	Cluster-28897.3248,UGT72E-2	0.98671772
	Cluster-28897.10573,PRDX6-7	0.982177139
	Cluster-51220.0,CAD-5	0.975532481
	Cluster-57653.0,COMT-2	0.956652884
	Cluster-28897.34522,PRDX6-5	0.952160164
	Cluster-28897.5566,PRDX6-8	0.943339199
	Cluster-28897.21232,CAD-6	0.919405397
Cluster-55249.0（bZIP-4）	Cluster-33784.0,PRDX6-1	0.998805144
	Cluster-28897.25631,bglX,bglB-1	0.997916582
	Cluster-28897.11757,PAL	0.989179189
	Cluster-28897.25617,bglX,bglB-5	0.988039931
	Cluster-28897.25635,bglX,bglB-6	0.984208342
	Cluster-28897.21343,bglX,bglB-10	0.950739077
	Cluster-28897.25627,bglX,bglB-3	0.9295974

	Cluster-28897.20416,PRDX6-2	-0.960991806
Cluster-28897.12451 (MYB46)	Cluster-28897.21461,PRDX6-4	-0.962897197
Cluster-28897.32290 (MYB3)	Cluster-28897.37941,bglX,bglB-8	0.993630074
	Cluster-14257.0,bglX,bglB-9	0.992665866
Cluster-28897.9562 (MYB)	Cluster-28897.37941,bglX,bglB-8	0.900361345
	Cluster-28897.16469,CAD-3	-0.936589512
Cluster-40484.0 (MYB1)	Cluster-45615.0,bglB	0.981268293
	Cluster-28897.5019,PRDX6-3	0.974612412
	Cluster-51096.0,COMT-3	0.972715218
	Cluster-28897.10004,UGT72E-1	0.961754095
	Cluster-28897.3248,UGT72E-2	0.961668942
	Cluster-51220.0,CAD-5	0.957575708
	Cluster-28897.10573,PRDX6-7	0.94228013
	Cluster-57653.0,COMT-2	0.912356917
Cluster-28897.12653 (NAC-1)	Cluster-28897.20416,PRDX6-2	0.94269443
	Cluster-28897.21230,CAD-2	-0.945353594
Cluster-28897.14163 (NAC-2)	Cluster-52394.0,bglX,bglB-7	0.980013493
	Cluster-28897.21461,PRDX6-4	0.938672294
	Cluster-28897.12824,REF1	0.903928132
	Cluster-28897.5861,COMT-1	-0.917561574
	Cluster-28897.3521,bglX	-0.946384642
	Cluster-28897.25606,bglX,bglB-2	-0.958303707
Cluster-28897.31142 (NAC-3)	Cluster-28897.25638,bglX,bglB-4	0.96513875
	Cluster-28897.25606,bglX,bglB-2	0.948604281
	Cluster-28897.21461,PRDX6-4	-0.905730978
	Cluster-28897.16469,CAD-3	-0.94195953
Cluster-54224.0 (NAC-4)	Cluster-28897.37941,bglX,bglB-8	-0.966500245
	Cluster-14257.0,bglX,bglB-9	-0.970144575
Cluster-28897.13025 (WRKY28)	Cluster-28897.25638,bglX,bglB-4	0.989175879
	Cluster-28897.12905,CAD-1	0.953769779
	Cluster-28897.25606,bglX,bglB-2	0.947349414
	Cluster-28897.5861,COMT-1	0.924697366
Cluster-28897.14612 (WRKY46)	Cluster-52394.0,bglX,bglB-7	0.992510671
	Cluster-28897.21461,PRDX6-4	0.940841434

	Cluster-28897.12824,REF1	0.932071812
	Cluster-28897.3521,bglX	-0.920821679
	Cluster-28897.25606,bglX,bglB-2	-0.933750356
Cluster-28897.24911 (WRKY3)	Cluster-28897.18603,CCoAOMT	0.991687514
	Cluster-28897.12824,REF1	0.986072745
	Cluster-52394.0,bglX,bglB-7	0.938824183
Cluster-28897.27523 (WRKY42)	Cluster-28897.12824,REF1	0.968833129
	Cluster-52394.0,bglX,bglB-7	0.957915754
	Cluster-28897.18603,CCoAOMT	0.93879901
Cluster-28897.28116 (WRKY70-1)	Cluster-28897.16469,CAD-3	0.982236499
	Cluster-28897.21343,bglX,bglB-10	0.923781054
Cluster-28897.28117 (WRKY70-2)	Cluster-28897.21461,PRDX6-4	0.946822191
Cluster-28897.30849 (WRKY70-3)	Cluster-14257.0,bglX,bglB-9	0.97326152
	Cluster-28897.37941,bglX,bglB-8	0.969945609
Cluster-28897.35914 (WRKY)	Cluster-52394.0,bglX,bglB-7	0.993958941
	Cluster-28897.12824,REF1	0.942831958
	Cluster-28897.21461,PRDX6-4	0.928678438
	Cluster-28897.3521,bglX	-0.917873152
	Cluster-28897.25606,bglX,bglB-2	-0.924277021
Cluster-28897.6659 (WRKY13)	Cluster-28897.10573,PRDX6-7	0.996636473
	Cluster-28897.34522,PRDX6-5	0.995441309
	Cluster-28897.5566,PRDX6-8	0.992601211
	Cluster-28897.10004,UGT72E-1	0.988704117
	Cluster-57653.0,COMT-2	0.984437209
	Cluster-51096.0,COMT-3	0.978048268
	Cluster-28897.5019,PRDX6-3	0.971491203
	Cluster-28897.3248,UGT72E-2	0.970901356
	Cluster-45615.0,bglB	0.965319418
	Cluster-28897.21232,CAD-6	0.953199077
	Cluster-51220.0,CAD-5	0.945998689
	Cluster-19366.0,UGT72E-3	0.904455595
